# Nanobodies raised against monomeric ɑ-synuclein inhibit fibril formation and destabilize toxic oligomeric species

**DOI:** 10.1186/s12915-017-0390-6

**Published:** 2017-07-03

**Authors:** Marija Iljina, Liu Hong, Mathew H. Horrocks, Marthe H. Ludtmann, Minee L. Choi, Craig D. Hughes, Francesco S. Ruggeri, Tim Guilliams, Alexander K. Buell, Ji-Eun Lee, Sonia Gandhi, Steven F. Lee, Clare E. Bryant, Michele Vendruscolo, Tuomas P. J. Knowles, Christopher M. Dobson, Erwin De Genst, David Klenerman

**Affiliations:** 1 Department of Chemistry, University of Cambridge, Lensfield Road, Cambridge, CB2 1EW UK; 2 Zhou Pei-Yuan Center for Applied Mathematics, Tsinghua University, Beijing, 100084 China; 3 Wellcome Trust-Medical Research Council Cambridge Stem Cell Institute, University of Cambridge, Cambridge, CB2 1QR UK; 4 Department of Molecular Neuroscience, University College London, Institute of Neurology, Queen Square, London, WC1N 3BG UK; 5 Department of Veterinary Medicine, University of Cambridge, Madingley Road, Cambridge, CB3 0ES UK; 6 Present address: Healx Ltd., St John’s Innovation Centre, Cowley Road, Cambridge, CB4 0WS UK; 7 Present address: Astra Zeneca, Innovative Medicines Discovery Sciences Unit 310, Darwin Building, Cambridge Science Park, Milton Road, Cambridge, CB4 0WG UK; 8 Present address: Institute of Physical Biology, University of Düsseldorf, Universitätsstr. 1, 40225 Düsseldorf, Germany

**Keywords:** Protein aggregation, Amyloid toxicity, Neurodegeneration, Aggregation inhibitors, Antibody, Single-molecule fluorescence

## Abstract

**Background:**

The aggregation of the protein ɑ-synuclein (ɑS) underlies a range of increasingly common neurodegenerative disorders including Parkinson’s disease. One widely explored therapeutic strategy for these conditions is the use of antibodies to target aggregated ɑS, although a detailed molecular-level mechanism of the action of such species remains elusive. Here, we characterize ɑS aggregation in vitro in the presence of two ɑS-specific single-domain antibodies (nanobodies), NbSyn2 and NbSyn87, which bind to the highly accessible C-terminal region of ɑS.

**Results:**

We show that both nanobodies inhibit the formation of ɑS fibrils. Furthermore, using single-molecule fluorescence techniques, we demonstrate that nanobody binding promotes a rapid conformational conversion from more stable oligomers to less stable oligomers of ɑS, leading to a dramatic reduction in oligomer-induced cellular toxicity.

**Conclusions:**

The results indicate a novel mechanism by which diseases associated with protein aggregation can be inhibited, and suggest that NbSyn2 and NbSyn87 could have significant therapeutic potential.

**Electronic supplementary material:**

The online version of this article (doi:10.1186/s12915-017-0390-6) contains supplementary material, which is available to authorized users.

## Background

The aberrant aggregation of the protein ɑ-synuclein (ɑS) has been linked to the onset and progression of Parkinson’s disease (PD), dementia with Lewy bodies [1], PD dementia, multiple system atrophy, and related synucleopathies [2–4]. The histopathological characteristics of PD and its associated disorders include the presence of neuronal inclusions, for example, Lewy bodies and Lewy neurites, which are primarily composed of fibrillar ɑS [1, 5]. The deposition of ɑS in the nervous system follows a characteristic pattern [6], and the ability of aggregated species to propagate across the brain by a mechanism that is defined as prion-like is increasingly recognized [7]. In aqueous solution, monomeric ɑS self-assembles into amyloid fibrils resembling those that are deposited in the brain, and the aggregation process proceeds through the formation of intermediate species, including soluble oligomers, prior to fibril formation [8, 9]. Moreover, these oligomeric species, rather than mature amyloid fibrils, have increasingly been identified as the most highly neurotoxic forms of ɑS [10–16].

As a consequence of the central role of ɑS aggregation in PD and the neurotoxicity of its assemblies, immunotherapy against ɑS is being widely pursued as a potential disease-modifying strategy [17, 18]. Although passive immunization using antibodies targeting ɑS has shown promise in numerous in vitro and in vivo model systems, as well as in several clinical trials [19, 20], there is no effective treatment for PD and other synucleinopathies. In order to make significant advances in this objective, it is crucial to develop a detailed understanding of the molecular mechanism of action of ɑS-specific antibodies during the aggregation process of the protein.

Over the years, full-length antibodies and antibody fragments, such as scFvs, have been generated against different regions and different species of ɑS for either biophysical studies, target validation, or therapy (reviewed in De Genst et al. [21] and Bergstrom et al. [20]), and have been used in multiple reported studies [22–27].

Full-length ɑS consists of 140 amino acid residues divided into three distinct regions, namely a positively charged lipid-binding N-terminal segment comprising residues 1–60, a central hydrophobic segment consisting of residues 61–95, and a negatively-charged C-terminal region comprising residues 96–140 [28]. This latter region does not include the residues observed to be in the amyloid fibril core [29], although previous in vitro studies have shown that its deletion promotes ɑS aggregation [30], indicating its involvement in the aggregation process. Notably, antibodies directed to the C-terminal region of ɑS, or specific for oligomeric forms of ɑS, were reported both to suppress strongly its aggregation and to reduce cellular toxicity [24, 27].

We have previously described two single-domain fragments of camelid heavy-chain antibodies [31], termed ‘nanobodies’ [32], denoted NbSyn2 [33] and NbSyn87 [34], which bind to distinctive epitopes within the C-terminal region of monomeric ɑS (residues 137–140 and 118–131, respectively). These nanobodies, which were raised against and bind to monomeric ɑS, also recognize fibrillar forms of ɑS in which the C-terminal region is exposed [29], allowing the use of these nanobodies as biophysical probes to study the properties of ɑS fibrils [34]. Moreover, one of these nanobodies, NbSyn87, when expressed as a genetic fusion protein with a PEST proteasomal degradation tag, was found to reduce toxicity in cell-lines overexpressing ɑS as a result of the specific degradation of monomeric ɑS, thereby significantly reducing proteostatic stress [35].

In order to understand such phenomena in molecular detail, it is important to characterize the impact of nanobody binding on the overall ɑS aggregation process in vitro, and in particular its effect on the formation of the highly toxic oligomers that are linked to cellular damage. It is known from previous studies that the C-terminal region of ɑS is exposed in at least some of its aggregated intermediate forms, such as oligomers [36], as well as monomers and fibrils, and that targeting of this region by antibodies has been recognized to have protective effects [19]. Therefore, it is plausible that the nanobodies and other C-terminal-binding antibodies can affect the formation and properties of these oligomers.

Since the pre-fibrillar oligomers are generally transient and heterogeneous, and are typically present at very low concentrations relative to ɑS monomers, they are difficult to study by conventional bulk techniques. We have previously utilized single-molecule Förster resonance energy transfer (sm-FRET) measurements in order to characterize the oligomerization of ɑS in considerable detail [13, 37]. Using this technique, we identified two distinct types of ɑS oligomers that were populated sequentially prior to the formation of ɑS fibrils [13]. The two oligomer types had distinct FRET efficiency distributions and were therefore termed “low-FRET” and “high-FRET” oligomers [13]. The differences in their photophysical properties, as well as their different kinetics of formation, stabilities towards dissociation and enzymatic proteolysis, and toxicity to cells indicated that these two oligomer forms were structurally distinct intermediates generated in the process of the formation of ɑS fibrils [13, 38]. In particular, the initial assembly of low-FRET oligomers was followed by slow conformational conversion into high-FRET oligomers prior to fibril formation. The accumulation of high-FRET oligomers was associated with the highest level of cytotoxicity, pointing to this specific oligomer type as the most damaging species formed during ɑS aggregation.

In this study, we set out to characterize in detail the effects of NbSyn2 and NbSyn87 on the aggregation of ɑS in vitro. First, we investigated fibril formation using a combination of biophysical experiments to monitor the kinetics of this process, and compared the properties of the various resulting fibrillar aggregates. Second, we employed sm-FRET techniques to explore how the presence of NbSyn87 and NbSyn2 affects the formation of the low-FRET and high-FRET oligomers. We identified the ability of the nanobodies to inhibit the formation of ɑS fibrils and to destabilize toxic high-FRET oligomers of ɑS, and explored how the latter process affects oligomer-induced cytotoxicity.

## Results

### NbSyn2 and NbSyn87 inhibit the formation, maturation, and elongation of ɑS fibrils

We first investigated the effects of the ɑS-specific nanobodies, NbSyn87 and NbSyn2, on ɑS fibril formation. To determine whether or not the binding of nanobodies to the C-terminal region affects the aggregation propensity of ɑS, we initially performed bulk solution aggregation assays to monitor fibril formation by full-length wild-type (wt) ɑS using thioflavin T (ThT) fluorescence measurements to monitor the quantity of aggregated ɑS. In these experiments, ɑS at a concentration of 70 μM was aggregated in the absence or presence of 140 μM NbSyn2, NbSyn87, or a control nanobody, cAbHuL5g (NbHul5g) [39]; the latter is a lysozyme-specific nanobody [39] and is therefore not expected to bind to ɑS.

**Fig. 1:**
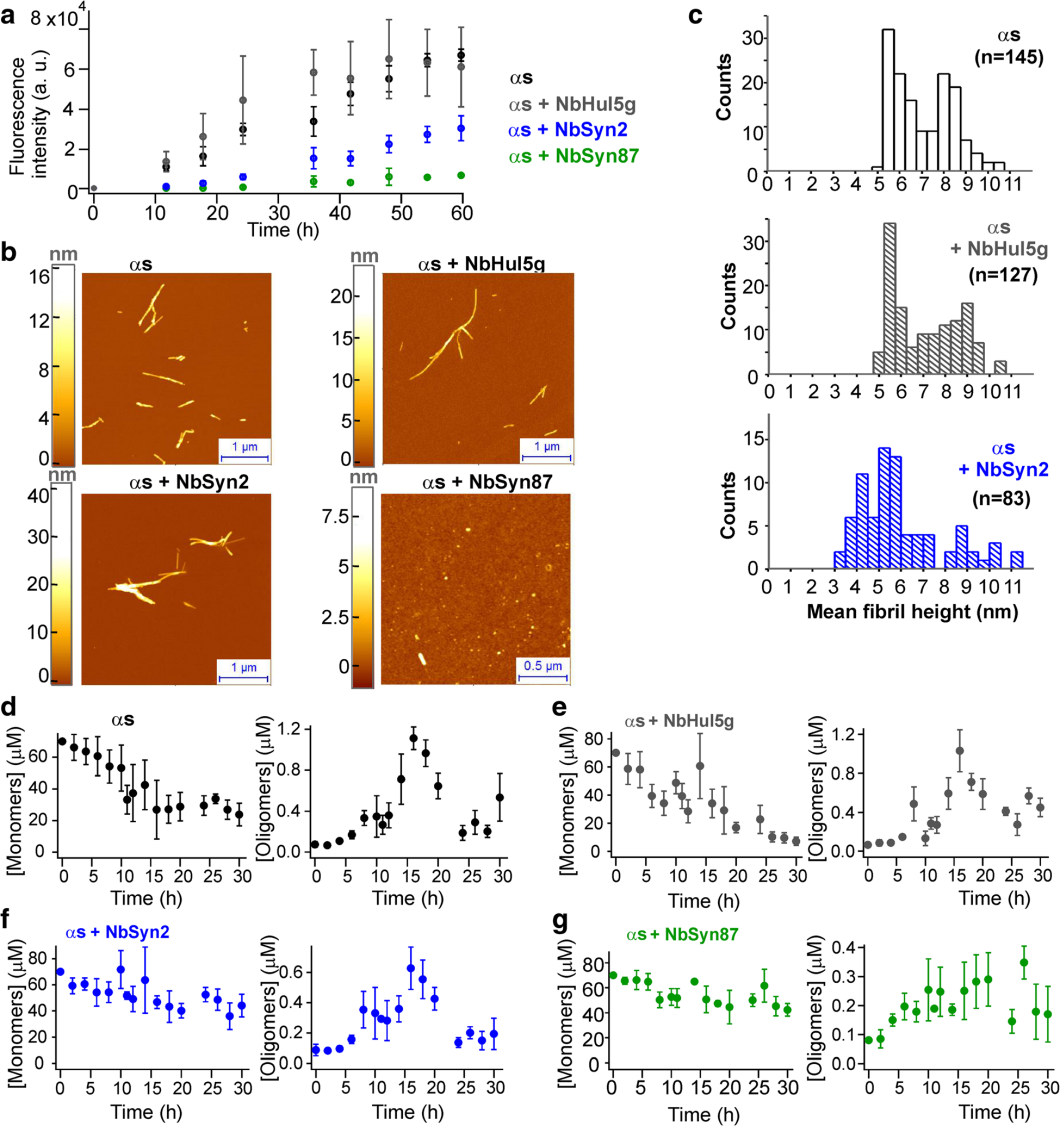
Aggregation kinetics of 70 μM ɑS in the absence and presence of nanobodies. **a** Bulk kinetic profiles of fibril formation, monitored by ThT fluorescence (n = 3, mean ± SEM). **b** Representative AFM images of the samples where the aggregation reaction had reached steady state (107 h); images of the samples prior to initiation of the aggregation reaction are shown in Additional file 1: Figure S1a. **c** Histograms representing the values of average fibril heights from AFM analysis. Scatter plots and statistical comparisons of these distributions are given in Additional file 1: Figure S1c. **d** sm-FRET kinetic profiles derived from monomer depletion (n = 6, mean ± SEM) and oligomer formation (at least n = 3, mean ± SEM) of ɑS at 70 μM in aqueous buffer, in the presence of 140 μM of the control NbHul5g (**e**), and in the presence of ɑS-specific nanobodies (**f** and **g**)

From Eq. 1 (Methods), taking the dissociation constants of NbSyn2 and NbSyn87 for monomeric ɑS, respectively, as approximately 264 nM and 42 nM [34] at 37 °C, we calculated that the free ɑS concentration under these conditions was approximately 260 nM and 40 nM, respectively, thereby approximating full saturation of all ɑS binding sites. The samples were incubated at physiologically relevant pH and ionic strengths (as detailed in the Methods section) at 37 °C with agitation to promote fibril formation; the resulting kinetic profiles of fibril formation are shown in Fig. 1a.

The aggregation kinetics of ɑS in the absence of nanobodies are in good agreement with our previously reported bulk kinetic measurements of ɑS under similar incubation conditions [13, 37]. In contrast, moderate inhibition of fibril formation was observed in the presence of NbSyn2, whereas solutions incubated in the presence of NbSyn87 gave rise to much lower ThT fluorescence signals over the entire duration of the experiment, indicating a stronger inhibition of ɑS fibril formation in the presence of this nanobody [13, 37]. In addition, we confirmed the inhibition of the formation of ThT-active aggregates in the presence of NbSyn2 and NbSyn87 by total internal reflection fluorescence microscopy imaging of the solutions extracted during the process of the aggregation reaction (Additional file 1: Figure S1).

Subsequently, we characterized the end products of aggregation using atomic force microscopy (AFM). In agreement with the findings from the ThT fluorescence assays, the images obtained in this way showed the presence of large numbers of amyloid fibrils in the solutions of ɑS that had been incubated in the absence of nanobodies or in the presence of NbHul5g or NbSyn2 (Fig. 1b). In the samples of ɑS incubated with NbSyn87, only small and apparently spherical aggregates along with monomeric species were observed, and no large fibrils could be detected (Fig. 1b). Comparison of average fibril heights from AFM maps revealed that fibrils formed in the presence of NbSyn2 were thinner than fibrils formed in the absence of nanobodies or in the presence of NbHul5g (Fig. 1c and Additional file 1: Figure S1), indicating that nanobodies impaired the maturation of ɑS fibrils. The differences in the fibril-forming capability of ɑS identified in the presence of the two ɑS-specific nanobodies might be due to the differences in the epitopes they bind. NbSyn87 binds with higher affinity and closer to the NAC region than does NbSyn2, and thereby might slow fibril formation due to steric hindrance and promote the formation of smaller fibrils (Fig. 1b).

We also performed quartz crystal microbalance experiments [40, 41] in order to measure the effects of NbSyn2 and NbSyn87 on the elongation rates of pre-formed ɑS fibrils upon their incubation with either monomeric ɑS, or with a stoichiometric ratio of monomeric ɑS and NbSyn87, NbSyn2, or NbHul5g (Additional file 2). It was found that the elongation rate of ɑS fibrils markedly decreased in the presence of both ɑS-specific nanobodies, corresponding to 30–40% of the rate in their absence, as described in further detail in Additional file 2: Tables S3 and S4, and discussed in Additional file 2: Supplementary Results.

### NbSyn2 and NbSyn87 impede the generation of high-FRET oligomeric species of ɑS prior to fibril formation

**Fig. 2:**
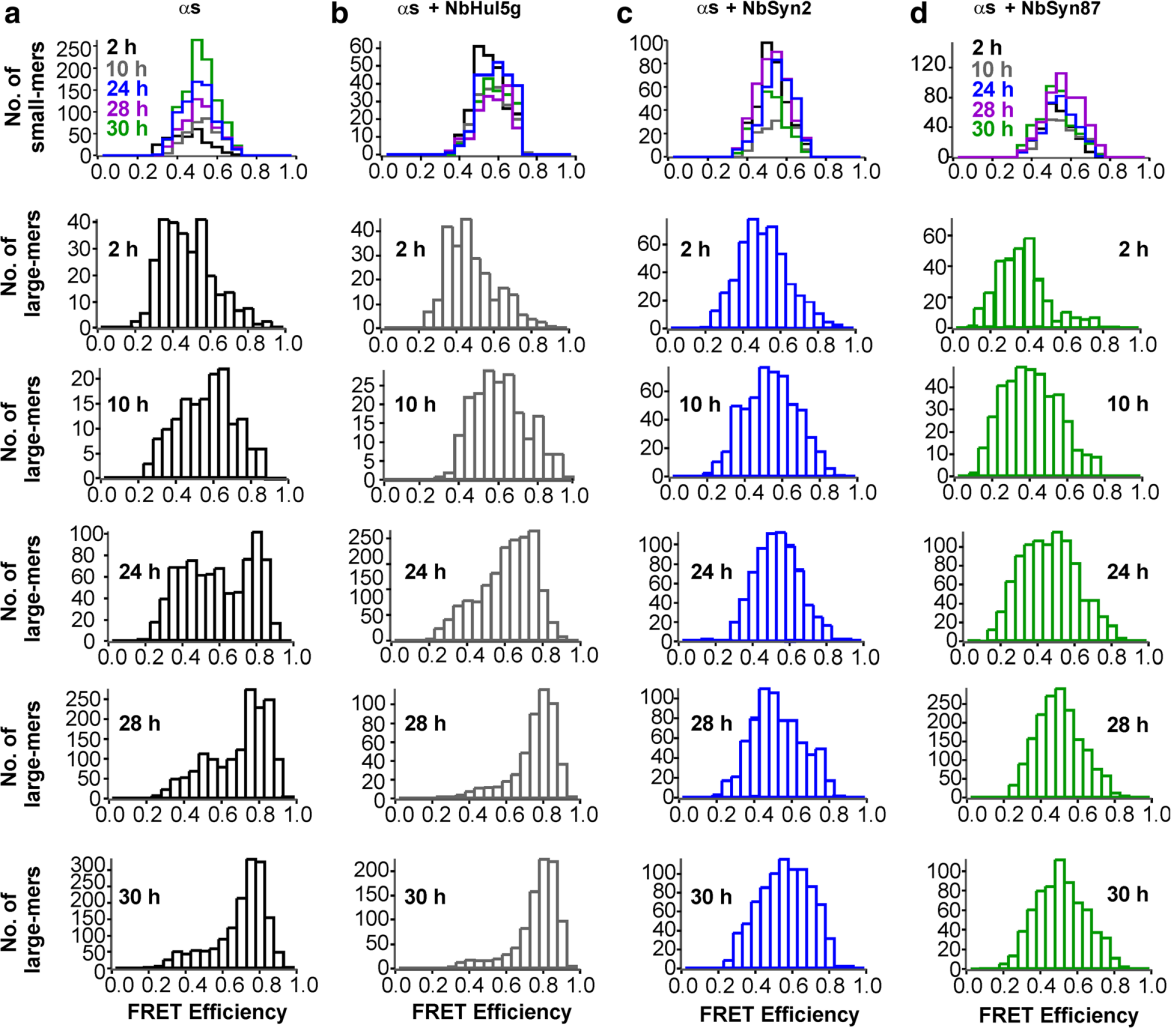
Representative FRET efficiency histograms obtained for different time points during the first 30 h of aggregation, recorded upon dilution into deionized water for sm-FRET detection. Here, the histograms have been split into two populations of apparent sizes, small-mers (2–5 monomer units) and large-mers (6–150 monomer units). For large-mers, the differences in the FRET efficiency histograms are clearly observable, especially at times longer than 24 h. In the histograms of ɑS-only and ɑS with NbHul5g, the peak centered at the FRET efficiency value of 0.8 is dominant and corresponds to high-FRET oligomers. In the presence of both ɑS-specific nanobodies, the histograms are centered at the FRET efficiency value of 0.5, and the maximum at 0.8 is absent

Having characterized the impact of the ɑS-specific nanobodies on the formation of ɑS fibrils, we subsequently set out to focus on the earlier steps in the ɑS aggregation reaction and to determine whether or not nanobodies affected oligomer formation. To this end, we used sm-FRET techniques developed in previous studies of the aggregation of ɑS in aqueous solution [13, 37, 38]. In sm-FRET experiments, an alanine to cysteine mutation at residue 90 of ɑS (A90C) was introduced to enable the attachment of a single Alexa Fluor (AF) dye; we have previously shown that this modification does not significantly influence the kinetics of ɑS fibril formation [13, 37, 38], a conclusion additionally confirmed in the present study by bulk ThT assays using wt and AF-labeled ɑS (Additional file 3: Figure S2a). Because residue 90 is located at the periphery of the central amyloidogenic region of the ɑS molecule [28], AF dyes come into close proximity during fibril formation. We have used AF488 and AF594 dyes, which we have found are sufficiently close together within the aggregates for FRET to be detectable, the efficiency of which is a useful readout of the aggregation reaction (Eq. 2, Methods). This approach makes it possible to detect two distinct oligomer types, those with a low FRET efficiency and those giving rise to a higher FRET efficiency, both of which are observed prior to fibril formation [13, 37, 38]. The sm-FRET analysis is selective for oligomeric aggregates and excludes large fibrillar species formed later during the aggregation process (as detailed in Methods and demonstrated in previous studies [13, 37, 38]). Therefore, this technique is complementary to the bulk ThT assay that has been used to monitor fibril formation (Fig. 1a). For every oligomer detected in the experiment, the FRET efficiency value and the apparent size in monomer units were calculated according to Eqs. 2 and 3 (Methods). These data are represented in Fig. 2 as histograms to show both the numbers and properties of the oligomer distributions.

In sm-FRET experiments, equimolar mixtures of AF488-labeled ɑS (AF488-ɑS) and AF594-labeled ɑS (AF594-ɑS) were used at a total protein concentration of 70 μM, either in the presence or absence of 140 μM of unlabeled nanobodies. The solutions were incubated with agitation under the same conditions as in the bulk ThT assays, and aliquots were withdrawn at regular time intervals during the incubation, diluted 10^5^-fold to generate appropriate conditions for single-molecule detection, and immediately analyzed by sm-FRET. We analyzed the solutions both following their dilution into aqueous buffer of the same composition as that used for the incubations (PBS, see in Methods), and following dilution into deionized water; we have recently shown that dilution into low ionic strength solutions enables an improved separation of the low- and high-FRET oligomers [38]. We measured the average brightness of AF488-ɑS and AF594-ɑS in the presence of the unlabeled nanobodies to verify that the nanobodies did not affect the fluorescence properties of the dyes in the sm-FRET experiments (Additional file 2: Supplementary Information). In addition, we carried out control experiments using samples where both ɑS and nanobodies were labeled with AF to confirm that the nanobodies were fully dissociated from ɑS during the detection step under both solution conditions; the results were consistent with previously reported K_d_ values [34], as detailed in Additional file 2: Supplementary Methods and Tables S1 and S2.

The resulting kinetic profiles of ɑS monomer depletion and oligomer formation are shown in Fig. 1d–g. The most rapid decrease in the monomer concentration, deduced from the monomer burst-rates [37], occurred in the samples containing 70 μM ɑS alone or in the presence of the control NbHul5g (Fig. 1d, e). However, samples containing NbSyn87 and NbSyn2 showed significantly slower monomer depletion. Based on the monomer depletion data derived from sm-FRET, after 30 h of aggregation, approximately 70% of the sample was aggregated in the ɑS-only reaction, 90% in the ɑS plus control nanobody, and approximately 40% in the samples containing both ɑS and NbSyn87 or NbSyn2. The oligomers were formed in all samples, and comprised less than 2% of the samples of ɑS (Fig. 1d–g).

Transmission electron microscopy (TEM) images of the samples at incubation times longer than 100 h confirmed the presence of abundant quantities of amyloid fibrils in the solutions containing ɑS alone or in the presence of NbHul5g and of NbSyn2, while only oligomers and short protofibrils were detected in the presence of NbSyn87 (Additional file 3: Figure S2b). These results confirm that the nanobodies inhibit ɑS fibril formation, and are therefore in good agreement with the bulk ThT fluorescence data and AFM results obtained for wt unlabeled ɑS as detailed above (Fig. 1b).

To elucidate the effects of the nanobodies on the formation of the low-FRET and high-FRET oligomers, individual FRET efficiency histograms of the samples recorded following dilution into water were analyzed. These showed clear differences between the control samples and the samples containing ɑS-specific NbSyn2 and NbSyn87 (Fig. 2). As in our previous work [13, 37], we separated the FRET efficiency histograms into two groups, corresponding to the histograms derived from “small” oligomers containing 2–5 apparent monomer units (denoted as “small-mers”) and oligomers containing 6–150 apparent monomer units (“large-mers”), based on the number of peaks that were resolvable in the FRET efficiency histograms, as previously reported [37]. This procedure allows us to identify the time-dependent changes readily, which are most clearly evident in the histograms for the large oligomers formed during aggregation (Fig. 2).

For the samples of ɑS alone, and ɑS in the presence of control NbHul5g, two peaks could be observed in the FRET efficiency histograms of the large-mers (Fig. 2), corresponding to the two previously identified oligomer populations [13, 37, 38]. Low-FRET oligomers are characterized by a population centered at an average FRET efficiency value (E) of 0.5, and high-FRET oligomers are indicated by the peak centered at E value of approximately 0.8 (Fig. 2). The high-FRET population was clearly distinguishable after 24 h of aggregation and was dominant by 30 h of incubation both in the absence of nanobodies and in the presence of the control nanobody (Fig. 2), in good agreement with our previously reported kinetics of high-FRET oligomer formation [13, 37]. The mild acceleration of the formation of high-FRET species in the presence of the control nanobody NbHul5g compared to the ɑS-only sample might be due to the crowding effect or transient interactions of NbHul5g with ɑS. Instead of exhibiting two populations at times longer than 24 h, however, the FRET histograms of oligomers formed in the presence of ɑS-specific nanobodies showed a single broad peak. The appearance of these histograms resembles those that had been obtained previously for ɑS solutions at low concentrations, in which high-FRET oligomers were not formed at high levels, yet were not fully absent [37]. Therefore, the appearance of the FRET efficiency histograms suggests that, in the presence of ɑS-specific nanobodies, the formation of high-FRET oligomers is impeded.

To characterize quantitatively the observed differences in ɑS oligomer populations either in the absence or presence of the nanobodies, we carried out a detailed kinetic analysis by fitting the experimental aggregation profiles to a nucleation-conversion-polymerization model similar to that reported previously [37]. A description of the analysis and the rate constants obtained for the individual microscopic steps of the aggregation process are available in Additional file 2: Supplementary Information and Additional file 4: Figure S3. We achieved the closest agreement with experimental data by assuming that both of the ɑS-specific nanobodies accelerate the reverse microscopic reaction steps, i.e., the conformational conversion from high-FRET oligomers to low-FRET oligomers and subsequently to monomers. This increase in rate results in the net inhibition of the overall forward aggregation process in the presence of NbSyn2 and NbSyn87, and successfully explains the experimentally observed reduced proportion of high-FRET oligomers and the altered kinetics of monomer depletion and fibril formation. Furthermore, this analysis predicted the distinct accumulation of low-FRET oligomers over longer aggregation times (Additional file 4: Figure S3). In the following section we discuss direct evidence for these phenomena.

### NbSyn2 and NbSyn87 enhance the conversion of high-FRET into low-FRET oligomeric species

Having characterized the kinetics of ɑS aggregation in the presence of NbSyn2 and NbSyn87, we set out to obtain direct evidence to support the conclusion that the nanobodies could promote the conformational conversion step from high-FRET to low-FRET species. To this end, we tested whether or not the addition of the nanobodies promoted the dissociation of pre-formed high-FRET oligomeric species. For these experiments, solutions from the aggregation reactions of 70 μM labeled ɑS in the absence of nanobodies were collected after 29 h of incubation and analyzed by sm-FRET to monitor the presence of high-FRET oligomers.

**Fig. 3:**
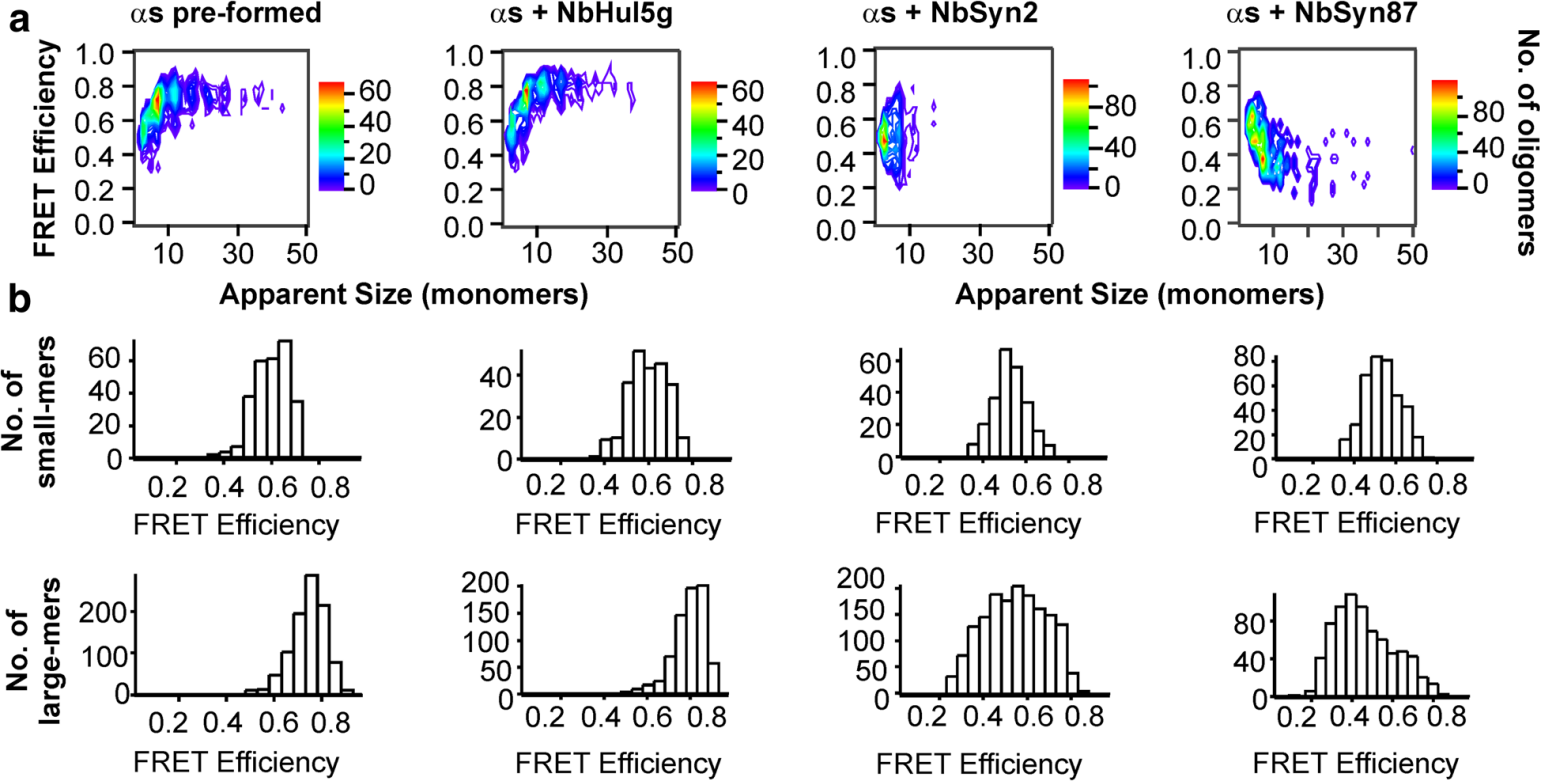
Results of sm-FRET measurements after addition of two equivalents of unlabeled nanobodies to pre-formed high-FRET oligomers. The oligomers were pre-formed by the aggregation of 70 μM ɑS without nanobodies for 29 h under the same incubation conditions as for the sm-FRET kinetic assays. **a** Representative contour plots of FRET efficiency versus apparent oligomer size resulting from sm-FRET measurements performed immediately after addition of nanobodies to aggregated solutions of ɑS (after 29 h). **b** Corresponding FRET efficiency histograms split into two size populations, small (2- to 5-mers) and large (6- to 150-mers) species. In the absence of nanobodies and upon addition of the control NbHul5g, the majority of the large oligomeric species observed in the experiment had FRET efficiency values above 0.6, characteristic of high-FRET oligomers (first two panels). Upon addition of either NbSyn2 (third panel) or NbSyn87 (fourth panel), the average FRET efficiency distributions shifted to lower values, an effect that was particularly pronounced in the presence of NbSyn87

Subsequently, two molar equivalents of unlabeled NbSyn2, NbSyn87 or NbHul5g were added to these samples prior to sm-FRET measurement. The representative contour plots of FRET efficiencies and apparent oligomer sizes, as well as the FRET efficiency histograms of each sample (Fig. 3) show highly reproducible results as nearly identical outcomes were observed in at least five independent experiments where fresh nanobodies were added to pre-formed high-FRET oligomers. The addition of NbSyn2 or NbSyn87 resulted in a reproducible decrease of the mean FRET efficiency values (Fig. 3b). These changes in FRET efficiencies cannot be explained by optical effects, e.g., quenching of fluorescence due to the binding of the nanobodies to the oligomeric species, because the samples were diluted by a factor of 10^5^, bringing the concentration of the NbSyn87 and NbSyn2 significantly below the Kd values of the interaction of the nanobodies with monomeric ɑS, as in all previous sm-FRET measurements [34]. In addition, the k_off_ values for NbSyn87 and NbSyn2 are the order of 0.01 and 0.1 s^–1^, respectively (Additional file 2: Supplementary Methods), indicating that the complexes would dissociate upon dilution, an assumption that was confirmed by the experimentally observed absence of coincidence between the labeled nanobody and ɑS under our detection conditions.

The changes in FRET efficiency values following the addition of NbSyn2 or NbSyn87 can therefore be attributed solely to the nanobody-induced conformational conversion of the high-FRET oligomers to the less-ordered low-FRET oligomers, in agreement with the results of the kinetic analysis. This conversion process was particularly fast in the presence of NbSyn87, with a rate constant of 1.0 ± 0.5 h^–1^ (Additional file 4: Figure S3); the more highly pronounced effect of NbSyn87 compared to NbSyn2 in this experiment is attributable to its higher affinity for ɑS. The oligomers will remain in the low-FRET structure during the analysis as the conformational reorganization from low-FRET to high-FRET oligomers in the absence of the nanobodies is characterized by a high energy barrier, reflected in a half-time of several hours [13].

The conformational conversion from high- to low-FRET oligomers described here was observed upon the addition of two molar equivalents of nanobodies. We also carried out titrations to determine the lowest stoichiometric ratio of the nanobodies to ɑS at which this behavior is observable. The reduction in FRET efficiencies in this experiment could be clearly observed down to 0.5 molar equivalents of the nanobodies, while at concentrations below 0.25 molar equivalents this process was too slow to be observed even upon incubation for 10 h (Additional file 5: Figure S4).

### The reduced population of high-FRET oligomers in the presence of NbSyn87 and NbSyn2 leads to significantly reduced cellular damage

The observation that NbSyn2 and NbSyn87 impair the formation of high-FRET oligomer types during the aggregation process and induce a more rapid conformational conversion of pre-formed high-FRET oligomers into low-FRET species, prompted us to test if these effects could result in an overall decrease in the average stability and cytotoxicity of the oligomers generated at the early stages of the aggregation process of ɑS.

**Fig. 4:**
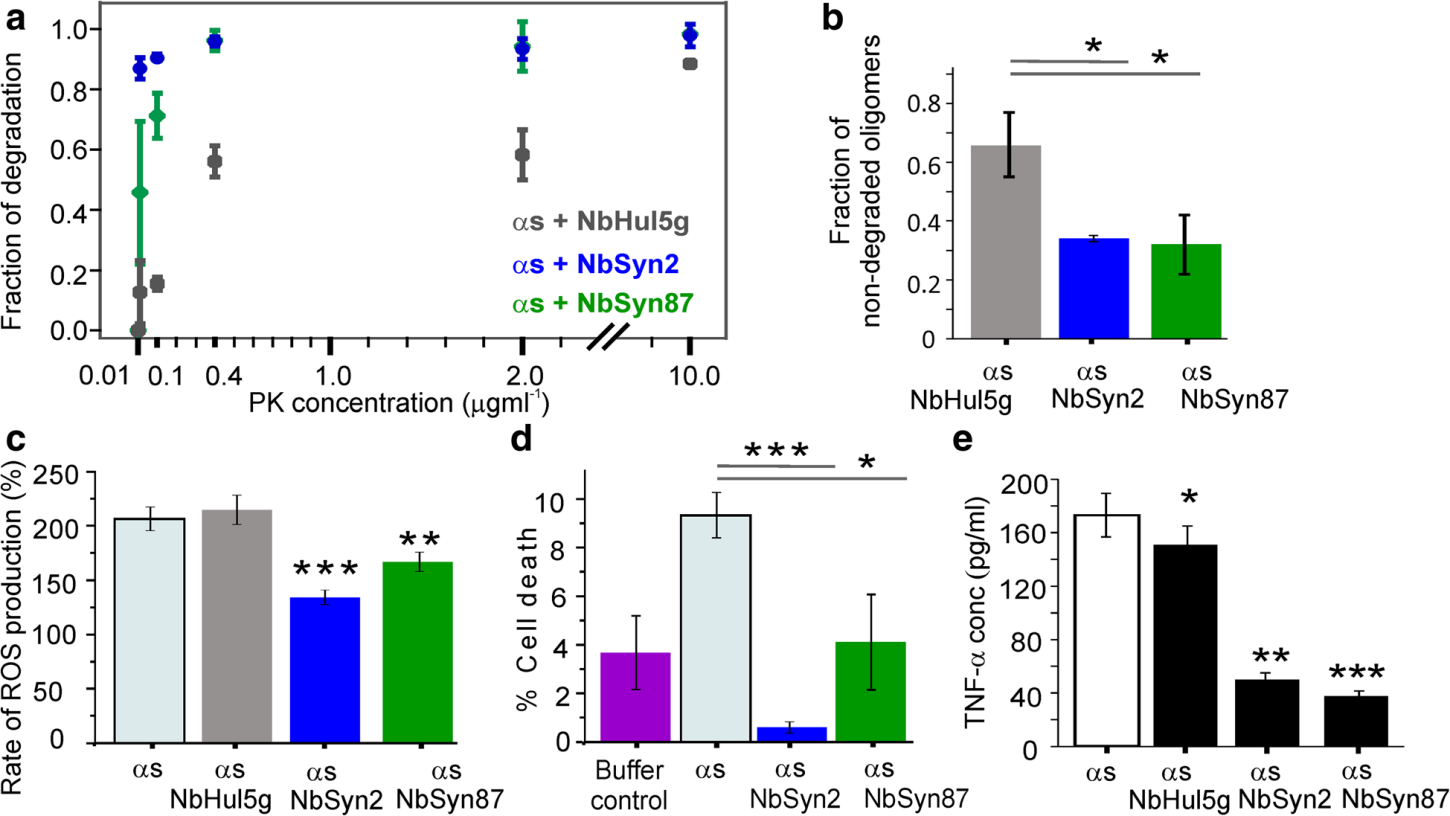
Comparative assays showing the relative stability and cytotoxicity of ɑS oligomers generated after aggregation for 29 h in the presence or absence of nanobodies under the same conditions as for the sm-FRET measurements. **a** Sm-FRET proteinase K digestion assays. The fraction of degradation is the number of oligomers in the proteinase K-exposed sample divided by the number of oligomers in the initial sample (n = 3, mean ± SD). Additional file 6: Figure S5 shows representative contour plots of FRET efficiencies versus apparent oligomer sizes. **b** Degradation of the 29 h samples by the 26S proteasome over 24 h (n = 3, SD, *P* < 0.05), analyzed by sm-FRET. **c** Cytoplasmic reactive oxygen species (ROS) production measured by monitoring the rate of dihydroethidium fluorescence (detailed in Additional file 2: Supplementary Methods). Application of ɑS oligomers to mixed cultures of rat hippocampal and cortical neurons (500 nM of total ɑS) induced an increase in the rate of ROS production relative to a control with no ɑS (taken as 100%); ɑS alone (n = 76 cells); ɑS with NbHul5g (n = 66 cells); ɑS with NbSyn2 (n = 89 cells, *P* < 0.001); and ɑS with NbSyn87 (n = 54 cells, *P* < 0.01). Additional file 8: Figure S6 shows representative plots of dihydroethidium fluorescence versus time. **d** Percentage cell death, measured by propidium iodide staining after incubation with the different ɑS oligomeric species. Cell death in the absence of ɑS (buffer control, n = 6 coverslips); upon incubation with ɑS alone (100 nM total ɑS, n = 7 coverslips); with ɑS with NbSyn2 (n = 5 coverslips, *P* < 0.001); with ɑS with NbSyn87 (n = 8 coverslips, *P* < 0.05). **e** Quantification of the released pro-inflammatory cytokine TNF-ɑ in BV2 microglia after the application of 29 h timepoints of ɑS (10 μM of the total ɑS), aggregated in the absence of nanobodies (n = 3, SEM), in the presence of 2 molar equivalents of NbHul5g (n = 3, SEM, *P* < 0.05), NbSyn2 (n = 3, SEM, *P* < 0.01), or NbSyn87 (n = 3, SEM, *P* < 0.001), and incubation with the cells for 24 h

It has previously been shown that high-FRET oligomers are more resistant towards digestion by proteinase K than are low-FRET oligomers, an observation that serves as an additional indication of the structural differences between these two oligomer types [13]. To determine whether or not the populations of oligomers formed in the presence of the nanobodies were more susceptible to proteinase K digestion than these formed in their absence, we exposed ɑS samples after 29 h of incubation under aggregating conditions to varying concentrations of proteinase K. The resulting digestion profiles are shown in Fig. 4a, and representative contour plots of FRET efficiencies and size distributions derived for individual samples in this assay are given in Additional file 6: Figure S5. From Fig. 4a, the populations of oligomeric species formed in the presence of NbSyn2 or NbSyn87 were more susceptible to proteinase K digestion than those formed in the presence of NbHul5g. This result is consistent with a lower proportion of more compact high-FRET oligomers in the solutions containing NbSyn2 and NbSyn87 than in the presence of NbHul5g, supporting the sm-FRET findings on the rates of interconversion. Subsequently, we measured whether or not the aggregates generated during the 29 h incubation period in the presence of ɑS-specific nanobodies were more degradable in comparison to the aggregates formed in the presence of the control nanobody by exposing these samples to the 26S proteasome for 24 h, and quantifying the numbers of non-degraded oligomers by sm-FRET, as described fully in Additional file 2: Supplementary Information. The results of this experiment indicate that the oligomer populations formed in the presence of the two specific nanobodies could be degraded by the proteasome to a greater extent than the aggregates formed in the presence of the control nanobody (Fig. 4b).

We then investigated if the inhibitory effect of the nanobodies at early stages of the aggregation reaction could result in a change in oligomer-induced cellular damage. High-FRET oligomers have been found in previous studies to induce significantly higher levels of cellular toxicity in rat mid-brain neuronal cultures than the low-FRET species [13, 37, 42]. Aliquots were extracted from samples of ɑS undergoing aggregation in the absence or presence of nanobodies after incubation for 29 h, and the production of reactive oxygen species (ROS) was measured following the application of these solutions to primary co-cultures of neurons and astrocytes (Additional file 2: Supplementary Information). The results show that the presence of NbSyn87 or NbSyn2 led to a significantly reduced rate of ROS production in comparison to the samples generated in their absence or in the presence of the control nanobody NbHul5g (Fig. 4c). This observed reduction in the oligomer-induced ROS indicates a reduced proportion of toxic high-FRET oligomers and supports the sm-FRET and proteinase K digestion results described above. We also investigated the ability of ɑS oligomer populations prepared in the presence or absence of nanobodies to induce cell death (see Additional file 2: Supplementary Information for details).

We found that the incubation of cells with ɑS aggregated in the presence of either NbSyn87 or NbSyn2 led to a significant reduction in cell death in comparison to the incubation of ɑS alone (Fig. 4d), indicating in the former the presence of a lower proportion of high-FRET compared to low-FRET oligomers. Apart from the significantly reduced cytotoxicity upon application of nanobody-bound oligomers in comparison to the oligomers of ɑS alone in ROS and cell death assays, it was found that the samples containing NbSyn2 reduced the cytotoxicity more effectively in comparison to NbSyn87 (Fig. 4c, d). While the overall reduction of cytotoxicity in comparison to the ɑS-only samples is entirely consistent with the measured ability of NbSyn87 and NbSyn2 to convert toxic high-FRET oligomers to low-FRET oligomers in aqueous solution, the difference between the two ɑS-specific nanobodies suggests the influence of other factors in the cell milieu in addition to the structure of ɑS oligomers. The lower protective effect of NbSyn87 compared to NbSyn2 in these experiments might be due to the high positive charge of NbSyn87 (pI > 9.0) and increased interaction with the cell membranes, as well as due to its ability to abolish ɑS fibril formation leading to the increased oligomer load. More detailed comparison of the reduction of toxicity by NbSyn2 and NbSyn87 in the cellular environment remains to be made in future studies.

Finally, we tested whether or not the oligomers formed in the presence of the ɑS-specific nanobodies reduced the pro-inflammatory activation of microglial cells by quantifying the concentration of the released pro-inflammatory cytokine, the tumor necrosis factor alpha (TNF-ɑ) protein [43], upon their incubation with ɑS solutions after aggregation for 29 h. The experimental procedure is described in Additional file 2: Supplementary Information, and the results show that the application of ɑS solutions aggregated with NbSyn2 and NbSyn87 resulted in a significantly reduced activation of microglia in comparison to ɑS alone and/or NbHul5g-containing samples (Fig. 4e).

Taken together, the comparative assays in Fig. 4 indicate a lower average stability of the ɑS oligomer populations generated in the presence of the ɑS-specific nanobodies and a reduced propensity to cause cellular damage. These findings are completely consistent with the conclusion that the population of the more toxic high-FRET oligomer type is reduced during the aggregation process of ɑS in the presence of NbSyn87 and NbSyn2.

## Discussion

In this study, we have characterized the key molecular steps that are altered by the ɑS-specific nanobodies, NbSyn2 and NbSyn87, during the aggregation reaction of ɑS. We have observed a clear effect of NbSyn2 and NbSyn87 on the formation of ɑS fibrils and their resulting morphology. In addition, using single-molecule fluorescence measurements we have identified a selective action of the nanobodies at the earliest stages of the aggregation reaction associated with the formation of toxic oligomeric species. In particular, both nanobodies were found to slow down the conformational conversion from less stable low-FRET oligomers to more stable high-FRET oligomers. Moreover, we observed that both NbSyn2 and NbSyn87 are able rapidly to convert pre-formed high-FRET oligomers to low-FRET species, indicating that the high-FRET oligomers are destabilized upon the binding of these nanobodies. These effects are associated with a significant reduction of cytotoxicity induced by the oligomers.

The ability of the nanobodies to promote the direct conformational conversion from more toxic to less toxic oligomeric species demonstrated in this study indicates the existence of an influential and unexplored mode of action of nanobodies or antibodies. Previously established modes of action of antibodies and nanobodies in vitro have included those that alter the equilibrium between fibrils, oligomers, and monomers [44], which can take a finite length of time to make sufficient changes in oligomer concentrations, and those that target oligomers to decrease their binding to cell membranes [11, 45]. The rapid conformational conversion by nanobodies of toxic oligomers to less toxic oligomers identified in our experiments has a direct effect on their ability to cause cellular damage. The results suggest, therefore, that the ability of nanobodies to promote conformational conversion of oligomers could have important potential for the development of therapeutic strategies. Since our study has been performed in vitro and at high concentrations of ɑS, it will be of interest to elucidate the role of this mechanism under a more complex biological environment in vivo in future investigations.

The ability of the nanobodies to destabilize high-FRET oligomers observed here may be due to a combination of their significantly smaller size than conventional antibodies [31] and their binding to the accessible C-terminal region of the protein [33, 34]. Antibodies binding to the C-terminal region of ɑS have previously been shown to decrease the production of highly aggregation-prone truncated forms of ɑS lacking the C-terminal region [46]. Thus, steric effects could be an important feature in enabling the smaller nanobodies to interact more effectively with oligomers than larger antibodies and further comparison of the action of nanobodies and conventional antibodies is warranted. Optimization of this destabilization effect in future work by probing the nanobody binding site in further detail may be possible through approaches that can distinguish between different oligomer structures, and hence measure the extent of destabilization.

## Conclusions

This study has involved the detailed biophysical characterization of the role of ɑS-specific nanobodies on the aggregation of ɑS in vitro, including the use of single-molecule fluorescence techniques. It shows that nanobodies targeting the C-terminal region inhibit the aggregation of ɑS not only by inhibiting its aggregation and elongation processes, but also by inhibiting the conformational conversion of oligomers formed prior to fibril formation. The latter effect leads to the decrease in oligomer stability and significantly reduces the cellular damage resulting from the effects of oligomeric species. Our work reveals an important mode of action of nanobodies, namely their ability rapidly to convert already formed toxic oligomers to less toxic ones. Such a mode of action has the potential to form the basis for a therapeutic strategy to combat PD and related protein misfolding conditions.

## Methods

### Expression and purification of nanobodies and ɑS

NbSyn87, NbSyn2, and NbHul5g were expressed in the periplasm of *E. coli* cells and purified using immobilized metal affinity chromatography and size-exclusion chromatography as previously described [33]. The amino acid sequences of NbSyn87 and NbSyn2 were as previously reported [34]. The sequence and structure of NbHul5g and the details of the construction of its more stable variant NbHul5g has been previously reported [39]. Expression and purification of wt and the alanine to cysteine mutant at position 90 (A90C) ɑS were carried out according to published protocols [47].

### ThT aggregation assays

Solutions of 600 μL of wt ɑS at a concentration of 70 μM either with or without 2 molar equivalents of NbSyn87, NbSyn2, or NbHul5g in PBS buffer (10 mM phosphate, pH 7.5, 100 mM NaCl, 2 mM EDTA, and 0.1% NaN_3_) were incubated for a period of 107 h at 37 °C with constant agitation at 200 rpm (New Brunswick Scientific Innova 43). Aliquots of 5 μL were removed at various time points and added to a 95 μL ThT solution (20 μM) in PBS buffer. ThT fluorescence was measured using a Cary Eclipse fluorimeter (Varian) with excitation and emission wavelengths of 440 and 480 nm, respectively. The mean maximum intensities from triplicate measurements were reported.

### AFM measurements

The end-point samples from the aggregation procedures used for the ThT assay were visualized using AFM imaging. Cleaved mica surfaces were incubated for 1 min with 10 μL of 0.5% (v/v) (3-aminopropyl)triethoxysilane (Sigma-Aldrich) in deionized water (Milli-Q). Then, the surfaces were rinsed three times with 1 mL of deionized water (Milli-Q) and dried with a gentle stream of nitrogen. For each sample, 10 μL of the solution was deposited on the positively functionalized surface. The droplet was incubated for 10 min, then rinsed with 1 mL of deionized water (Milli-Q) and dried with a gentle stream of nitrogen; all procedures were carried out at room temperature. AFM maps were acquired using JPK Nanowizard 2 system operating in tapping mode and equipped with a silicon tip (Micromasch, 2 Nm^–1^) with a nominal radius of 10 nm. Image flattening was performed by SPIP (Image Metrology) software. Analysis of mean fibril heights was carried out as previously reported [48] and described in detail in Additional file 2: Supplementary Information.

### ɑS labeling

The A90C mutant variant of ɑS was labeled with either maleimide-modified AF488 or AF594 dyes (Life Technologies) via the cysteine thiol moiety as previously reported [13, 49]. The labeled protein was purified from the excess of free dye using a P10 desalting column with Sephadex G25 matrix (GE Healthcare) and concentrated using Amicon Ultra Centricons (Millipore), divided into aliquots, flash frozen and stored at −80 °C. Each aliquot was thawed immediately and used only once.

### Estimation of the concentration of free ɑS

The concentration of free ɑS was estimated as the difference between the total starting concentration of ɑS and the concentration of bound ɑS in the presence of nanobodies, calculated according to:

1 $$ {\left[ as\right]}_{bound}=\frac{1}{2}\left(\left[ as\right]+\left[ Nb\right]+{K}_d-\sqrt{{\left(\left[ as\right]+\left[ Nb\right]+{K}_d\right)}^2-4\left[ as\right]\left[ Nb\right]}\right) $$

where [*as*]_*bound*_ is the concentration of bound ɑS, [*as*] and [*Nb*] are the starting concentrations of ɑS and nanobody, and *K*_*d*_ is the corresponding dissociation constant [34].

### Sm-FRET experiments

The experiments were carried out according to previously published procedures [37, 38], utilizing a custom-built single-molecule setup as already described [50] and microfluidic devices fabricated as previously reported [38]. The single-molecule instrumentation has been previously described in detail [37]. For the aggregation reactions, equimolar concentrations of AF488- and AF594-labeled A90C ɑS (AF488-ɑS and AF594-ɑS) in PBS were combined to a final volume of 300 μL, bringing the total ɑS concentration to 70 μM, either in the presence or absence of 140 μM of unlabeled NbSyn87, NbSyn2, or NbHul5g. At least three separate samples were prepared and analyzed at each protein combination. The solutions were incubated in the dark for up to 50 h under the same incubation conditions as the samples for ThT assays (37 °C, with agitation). Aliquots were withdrawn at regular time intervals and diluted 2.5 × 10^5^ fold by serial dilution in either PBS buffer or deionized water (Milli-Q) to give a concentration suitable for analysis in the single molecule regime. Immediately upon dilution, the solution was passed at a constant rate of 2 cm s^–1^ (syringe pump PHD2000, Harvard Apparatus) through a channel of a microfluidic device mounted on the single-molecule confocal microscope (Nikon Eclipse Ti-U). The 488 nm laser beam was focused into the center of the channel (10 μm along vertical direction). The experimental data consisted of synchronous time-binned fluorescence output from the donor (AF488) and the acceptor (AF594) channels, acquired for 400 s per aliquot (80 frames, 100,000 bins frame^–1^, 50 μs bin-width). The absence of oligomer dissociation during sm-FRET detection was previously confirmed [37], and the estimated oligomer concentrations were previously found to agree with the oligomer concentrations at bulk conditions [13].

### Sm-FRET data analysis

The resulting sm-FRET data were analyzed as previously reported [37, 38], using custom-written Igor Pro code (Wavemetrics). Time-bins with intensities above 15 photons.bin^–1^ were selected in both the donor and acceptor channel (AND criterion) [51]. Simultaneous fluorescence bursts above the threshold were assigned to oligomeric events and the non-simultaneous donor bursts above the threshold correspond to monomeric ɑS. The intensities of the selected photon bursts were corrected for the crosstalk from donor to acceptor channel (13%), and the autofluorescence in the acceptor channel (1.3 photons.bin^–1^). For each oligomeric burst, the two key parameters calculated were the apparent size of the oligomer (*Size*) and the FRET efficiency (*E*), defined as:

2 $$ E=\frac{I_A}{I_A+\gamma {I}_D} $$

3 $$ Size=2\times \frac{I_D+{\gamma}^{-1}{I}_A}{I_{monomer}} $$

where *I*_*D*_ is the donor intensity in the presence of an acceptor, *I*_*A*_ is the acceptor intensity, $\gamma$ is the gamma factor, which corrects for the differences in detection efficiencies of the two fluorescent probes and their quantum yields ($\gamma$ = 0.99), and *I*_*monomer*_ is the mean monomer brightness, averaged over all of the non-coincident bursts in the donor channel. FRET efficiency and apparent size distributions were represented as histograms with bin-widths of 0.05 and 1, respectively. The presence of undetectable singly-colored or fluorescently quenched oligomers was assumed to have no significant effect on the data, based on our previous works [13, 38]. Large species, either those apparently greater than 150 monomers in size or occupying consecutive time-bins, were assumed to be due to fibrillar species and were excluded from the analysis, as reported in our previous study [38]. Subsequently, both the monomer and the oligomer bursts were converted into corresponding bulk ɑS concentrations by taking into account the dilution factor, as previously reported [37].

## Additional files

Additional file 1: Figure S1. Additional results. (**a**) AFM images of starting monomeric solutions of wild-type (wt) ɑS prior to incubation with agitation. (**b**) Total internal reflection fluorescence microscopy results. Left: representative sum-image in the ThT emission channel (100 frames) and the corresponding reconstruction image in the NR channel (2000 frames). Right: comparison of the total numbers of aggregates and percent of ThT-active aggregates formed in the wt ɑS-only and wt ɑS + nanobody solutions at the same time-point of the aggregation process. (**c**) Scatter plots and statistical comparison of the distributions of average fibril heights derived from AFM maps (Fig. 1b, c, main text). (**d**) Quartz crystal microbalance recordings using ɑS (21 μM), nanobody alone (21 μM) or 1:1 mixture of ɑS with nanobody (21 μM : 21 μM) or control peptide (42 μM). (TIF 4648 kb)

Additional file 2: Supplementary Information. (PDF 462 kb)

Additional file 3: Figure S2. Results of control bulk ThT experiments and TEM imaging of labeled ɑS. (**a**) Progression of fibril formation, monitored by ThT fluorescence emission from either unlabeled wild-type (black) or AF-594 labeled ɑS at 70 μM (n = 3, SD). (**b**) TEM images of aggregates formed in 70 μM 1:1 AF488:AF594 dual-labeled ɑS solutions. Top: labeled ɑS solutions at different time-points during aggregation. Bottom: ɑS solutions in the presence of 140 μM of unlabeled NbHul5g, NbSyn2, and NbSyn87 after more than 100 h incubation with agitation. Large amyloid fibrils and fibrillar fragments were observed in all samples at this time, except in the presence of NbSyn87, where short protofibrils were present along with oligomeric aggregates. (TIF 4331 kb)

Additional file 4: Figure S3. Kinetic analysis of ɑS aggregation. (**a**) Schematic representation of the nucleation-conversion-polymerization model. Monomeric ɑS form low-FRET oligomers with rate constant *k*_*n*_ and an average reaction order *n*_*c*_. Low-FRET oligomers convert into high-FRET oligomers by a first-order reaction with a rate constant *k*_1_^*c*^, which is followed by a first-order conversion to fibrils, with a rate constant *k*_2_^*c*^. Once formed, fibrils grow by monomer addition with a length-independent rate constant *k*_*e*_. The conversion steps between oligomer types are treated as size independent, and *k*_1_^*c*^ is set to be equal to *k*_2_^*c*^. First-order reverse conversion reactions from high-FRET to low-FRET oligomers and from low-FRET oligomers to monomers are introduced, with rate constants $\tilde{{\mathrm{k}}_1^{\mathrm{c}}}$ and $\tilde{{\mathrm{k}}_{\mathrm{n}}}$, respectively. (**b**–**e**) This model was fitted globally to the kinetic data of ɑS aggregation at 70 μM in the absence or the presence of 140 μM of nanobodies. The global fits (dashed lines) were performed with parameters *k*_*n*_ = (1.0 ± 0.5) × 10^− 3^ h^− 1^, *k*_*e*_ = 0.16 ± 0.08 μM^− 1^h^− 1^, *k*_1_^*c*^ = *k*_2_^*c*^ = 0.12 ± 0.04 h^− 1^, $\tilde{{\mathrm{k}}_{\mathrm{n}}}=\tilde{{\mathrm{k}}_1^{\mathrm{c}}}=0\;{\mathrm{h}}^{-1}$, *n*_*c*_ = 1 ± 0.1, *m*_*tot*_ = 70 μM for reactions containing ɑS only and ɑS with NbHul5g. The reverse reaction from high-FRET oligomers to low-FRET oligomers was found to be accelerated in the presence of NbSyn2 and NbSyn87, with $\tilde{{\mathrm{k}}_{\mathrm{n}}}=0.2\pm 0.1\;{\mathrm{h}}^{-1}$ and $\tilde{{\mathrm{k}}_1^{\mathrm{c}}}=1\pm 0.5\;{\mathrm{h}}^{-1}$ for ɑS aggregation in the presence of NbSyn2, and with $\tilde{{\mathrm{k}}_{\mathrm{n}}}=0.25\pm 0.1\;{\mathrm{h}}^{-1}$ and $\tilde{{\mathrm{k}}_1^{\mathrm{c}}}=10\pm 5\;{\mathrm{h}}^{-1}$ for ɑS aggregation in the presence of NbSyn87, with all remaining parameters unchanged. (**f**) Predictions of the concentrations of low-FRET and high-FRET oligomers during 100 h of ɑS aggregation in the presence or absence of nanobodies. (TIF 710 kb)

Additional file 5: Figure S4. Representative FRET efficiency histograms from the ‘reverse’ sm-FRET experiments, analogous to those shown in Fig. 3 (main text). (**a**) Pre-formed high-FRET oligomers were formed in a forward incubation of monomeric ɑS (70 μM in PBS, 29 h, shaking at 37 °C). To the pre-formed oligomer solutions, either 1, 0.5, or 0.25 molar equivalents of nanobodies, NbSyn87 (**b**) or NbSyn2 (**c**), were added and sm-FRET detection was carried out within 5 min after the addition. In the case of 0.25 equivalents, the samples were further incubated at 37 °C under quiescent conditions (in low-binding test-tubes), and sm-FRET analysis repeated. (TIF 877 kb)

Additional file 6: Figure S5. Representative contour plots of FRET efficiency and size after proteinase K digestion of 29-h time-points by different concentrations of proteinase K (Fig. 4a, main text). (**a**) Control sample containing NbHul5g is less degradable in comparison to the samples prepared in the presence of NbSyn2 (**b**) and NbSyn87 (**c**), as indicated by the presence of higher oligomer fraction remaining in the sample upon incubation with proteinase K. This is consistent with the presence of high-FRET oligomers in the sample. (TIF 1028 kb)

Additional file 7: Supporting Data Values. (XLSX 27 kb)

Additional file 8: Figure S6. Representative result from the reactive oxygen species measurements presented in main text, Fig. 4c. Application of 500 nM of AF-labeled ɑS solution induced an increase in the ratio of dihydroethydium (HEt) fluorescence between its oxidized and non-oxidized forms. The time when ɑS was applied is marked with the grey bracket on the plot. A higher increase in HEt ratio is observed upon application of ɑS solutions containing control NbHul5g, suggesting that oligomers formed in its presence are more damaging in comparison to the oligomers formed in the presence of ɑS-specific nanobodies. (TIF 108 kb)
