# Supplementary figures and images for: Nanobodies raised against monomeric ɑ-synuclein inhibit fibril formation and destabilize toxic oligomeric species
Source: BMC Biol. 2017 Jul 3;15:57. doi: 10.1186/s12915-017-0390-6 (PMC5496350; doi:10.1186/s12915-017-0390-6)

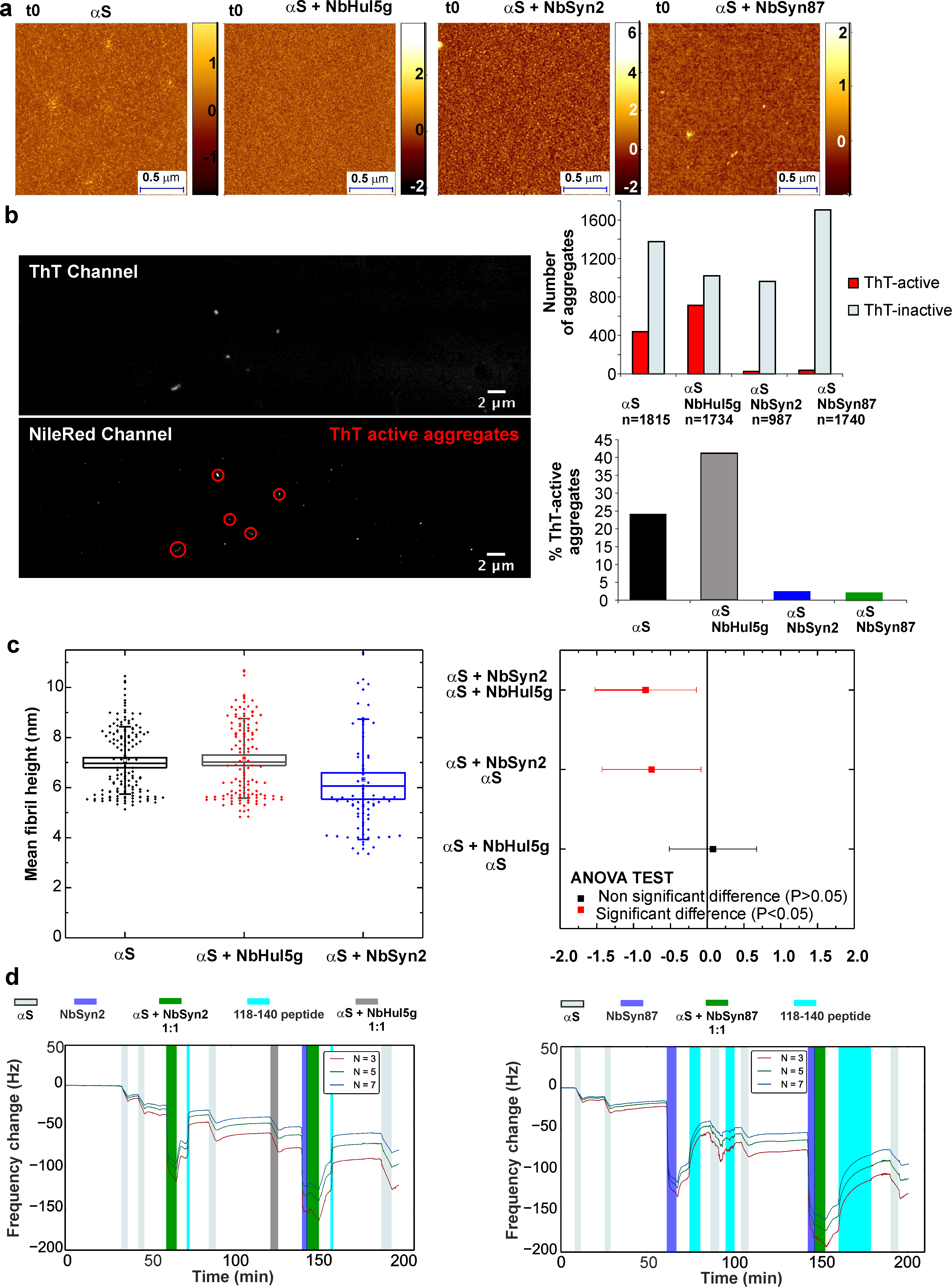

Supplement: Supplementary file 1 — Additional results. (a) AFM images of starting monomeric solutions of wild-type (wt) ɑS prior to incubation with agitation. (b) Total internal reflection fluorescence microscopy results. Left: representative sum-image in the ThT emission channel (100 frames) and the corresponding reconstruction image in the NR channel (2000 frames). Right: comparison of the total numbers of aggregates and percent of ThT-active aggregates formed in the wt ɑS-only and wt ɑS + nanobody solutions at the same time-point of the aggregation process. (c) Scatter plots and statistical comparison of the distributions of average fibril heights derived from AFM maps (Fig. 1b, c, main text). (d) Quartz crystal microbalance recordings using ɑS (21 μM), nanobody alone (21 μM) or 1:1 mixture of ɑS with nanobody (21 μM : 21 μM) or control peptide (42 μM). (TIF 4648 kb) [file 12915_2017_390_MOESM1_ESM.tif]

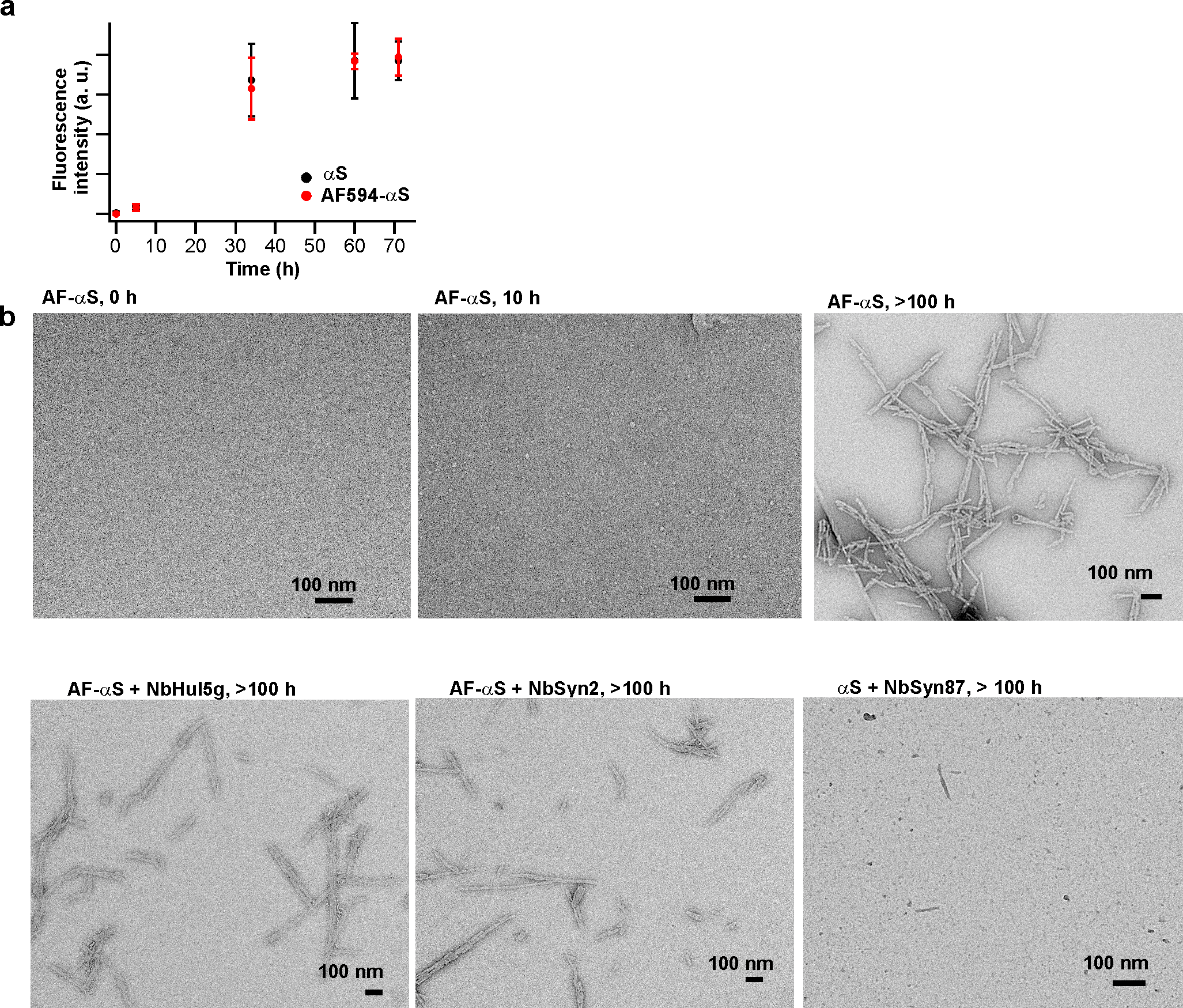

Supplement: Supplementary file 3 — Results of control bulk ThT experiments and TEM imaging of labeled ɑS. (a) Progression of fibril formation, monitored by ThT fluorescence emission from either unlabeled wild-type (black) or AF-594 labeled ɑS at 70 μM (n = 3, SD). (b) TEM images of aggregates formed in 70 μM 1:1 AF488:AF594 dual-labeled ɑS solutions. Top: labeled ɑS solutions at different time-points during aggregation. Bottom: ɑS solutions in the presence of 140 μM of unlabeled NbHul5g, NbSyn2, and NbSyn87 after more than 100 h incubation with agitation. Large amyloid fibrils and fibrillar fragments were observed in all samples at this time, except in the presence of NbSyn87, where short protofibrils were present along with oligomeric aggregates. (TIF 4331 kb) [file 12915_2017_390_MOESM3_ESM.tif]

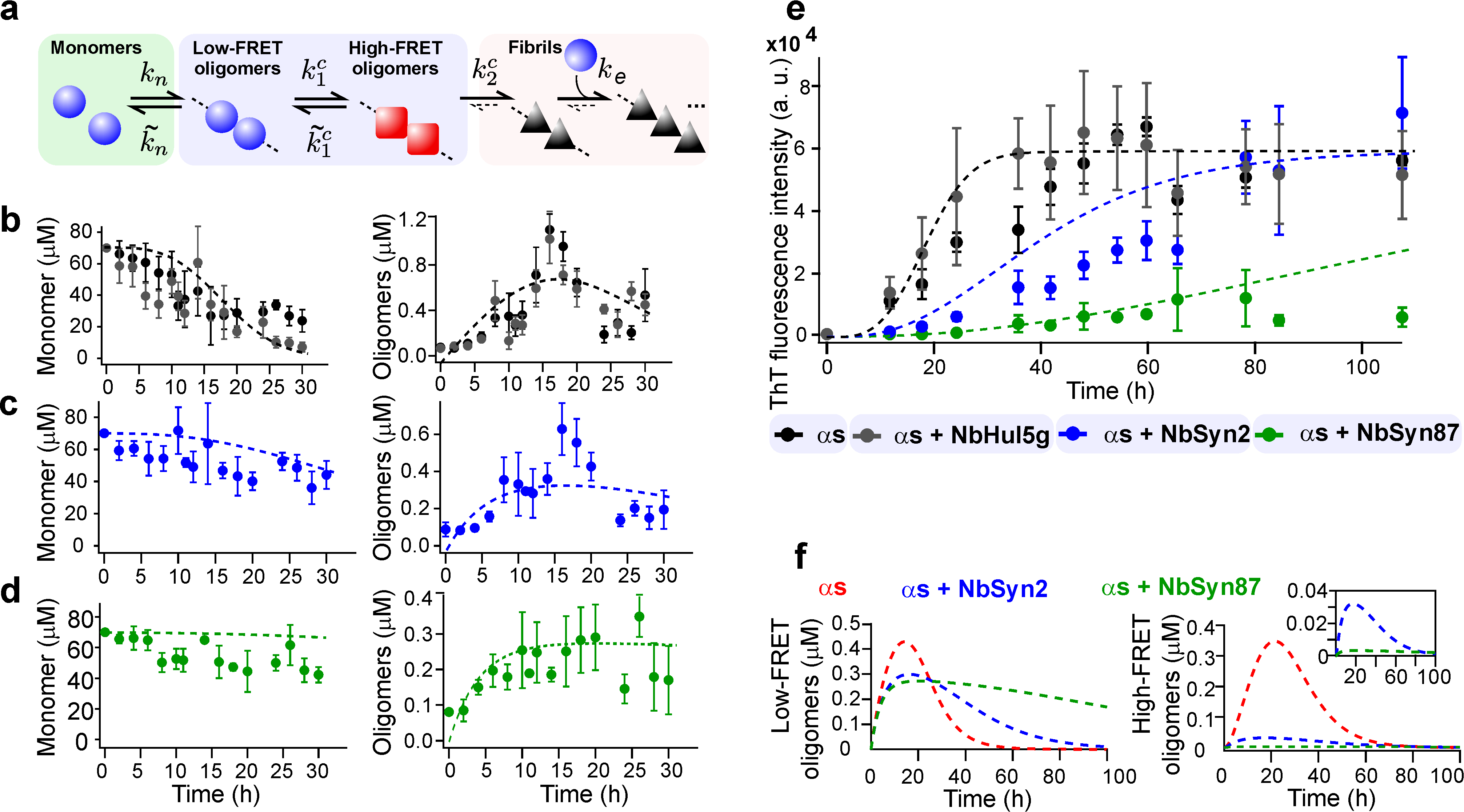

Supplement: Supplementary file 4 — Kinetic analysis of ɑS aggregation. (a) Schematic representation of the nucleation-conversion-polymerization model. Monomeric ɑS form low-FRET oligomers with rate constant k n and an average reaction order n c. Low-FRET oligomers convert into high-FRET oligomers by a first-order reaction with a rate constant k 1 c, which is followed by a first-order conversion to fibrils, with a rate constant k 2 c. Once formed, fibrils grow by monomer addition with a length-independent rate constant k e. The conversion steps between oligomer types are treated as size independent, and k 1 c is set to be equal to k 2 c. First-order reverse conversion reactions from high-FRET to low-FRET oligomers and from low-FRET oligomers to monomers are introduced, with rate constants $\tilde{{\mathrm{k}}_1^{\mathrm{c}}}$k1c˜ and $\tilde{{\mathrm{k}}_{\mathrm{n}}}$kn˜, respectively. (b–e) This model was fitted globally to the kinetic data of ɑS aggregation at 70 μM in the absence or the presence of 140 μM of nanobodies. The global fits (dashed lines) were performed with parameters k n = (1.0 ± 0.5) × 10− 3 h− 1, k e = 0.16 ± 0.08 μM− 1h− 1, k 1 c = k 2 c = 0.12 ± 0.04 h− 1, $\tilde{{\mathrm{k}}_{\mathrm{n}}}=\tilde{{\mathrm{k}}_1^{\mathrm{c}}}=0\;{\mathrm{h}}^{-1}$kn˜=k1c˜= [file 12915_2017_390_MOESM4_ESM.tif]

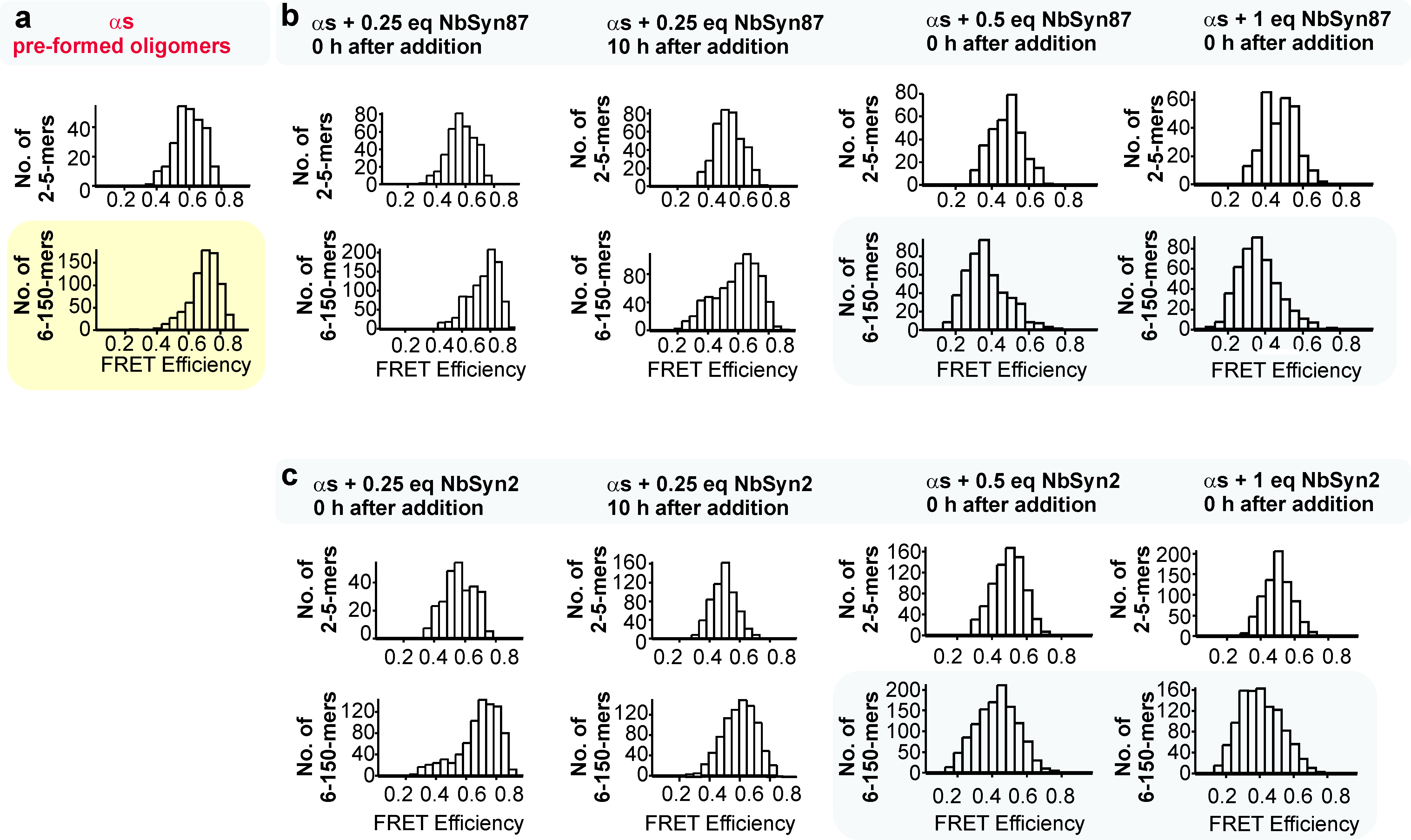

Supplement: Supplementary file 5 — Representative FRET efficiency histograms from the ‘reverse’ sm-FRET experiments, analogous to those shown in Fig. 3 (main text). (a) Pre-formed high-FRET oligomers were formed in a forward incubation of monomeric ɑS (70 μM in PBS, 29 h, shaking at 37 °C). To the pre-formed oligomer solutions, either 1, 0.5, or 0.25 molar equivalents of nanobodies, NbSyn87 (b) or NbSyn2 (c), were added and sm-FRET detection was carried out within 5 min after the addition. In the case of 0.25 equivalents, the samples were further incubated at 37 °C under quiescent conditions (in low-binding test-tubes), and sm-FRET analysis repeated. (TIF 877 kb) [file 12915_2017_390_MOESM5_ESM.tif]

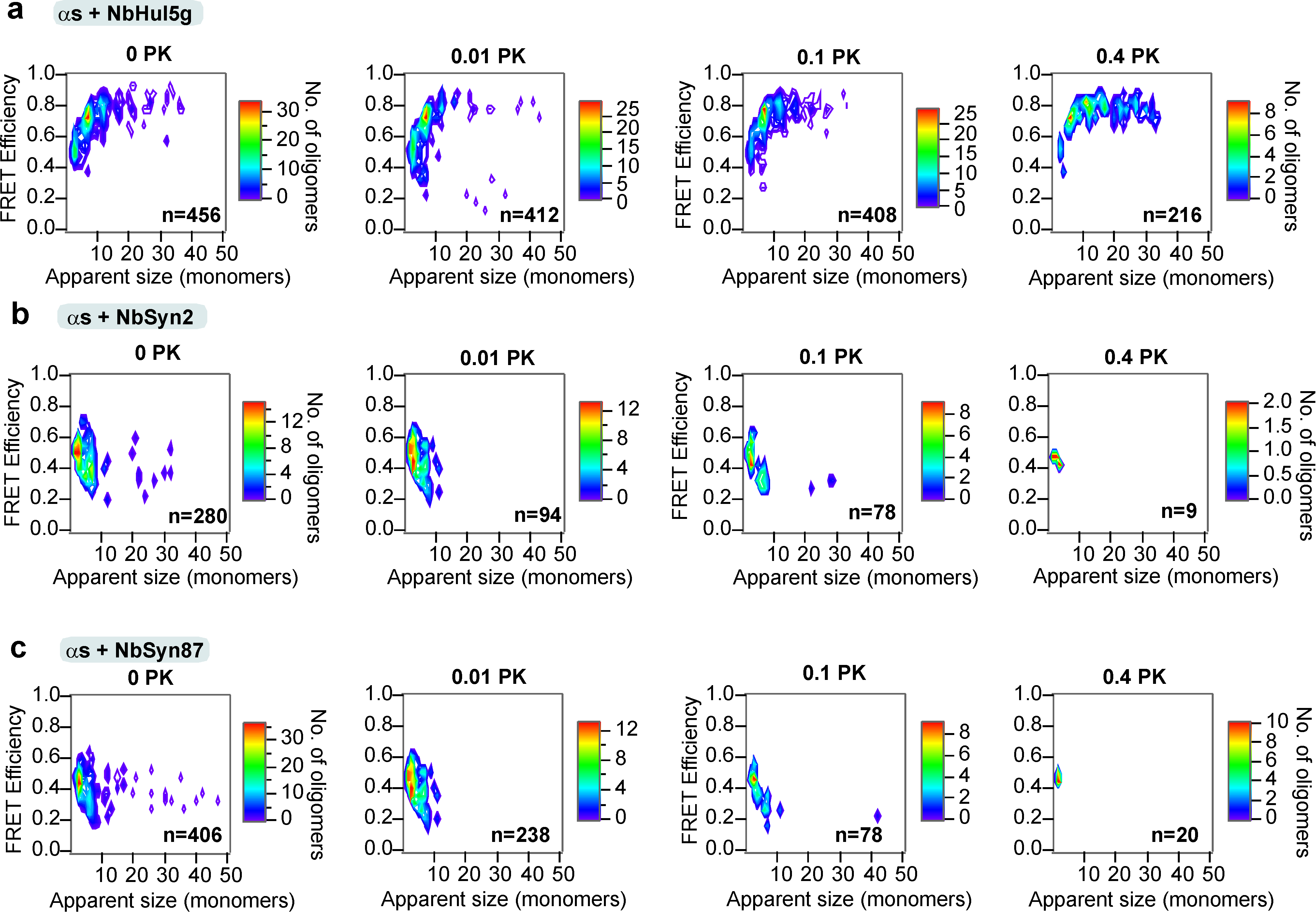

Supplement: Supplementary file 6 — Representative contour plots of FRET efficiency and size after proteinase K digestion of 29-h time-points by different concentrations of proteinase K (Fig. 4a, main text). (a) Control sample containing NbHul5g is less degradable in comparison to the samples prepared in the presence of NbSyn2 (b) and NbSyn87 (c), as indicated by the presence of higher oligomer fraction remaining in the sample upon incubation with proteinase K. This is consistent with the presence of high-FRET oligomers in the sample. (TIF 1028 kb) [file 12915_2017_390_MOESM6_ESM.tif]

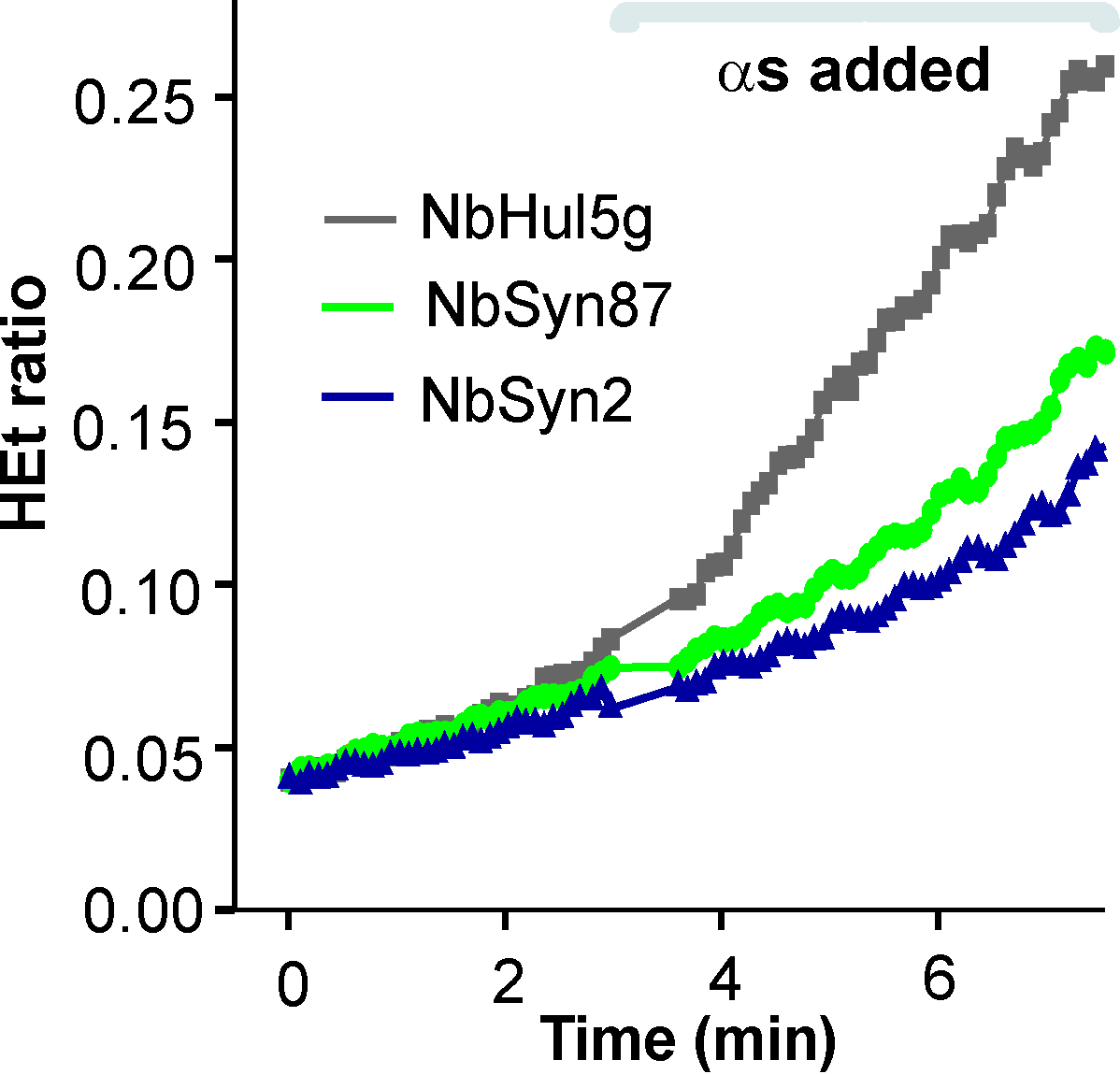

Supplement: Supplementary file 8 — Representative result from the reactive oxygen species measurements presented in main text, Fig. 4c. Application of 500 nM of AF-labeled ɑS solution induced an increase in the ratio of dihydroethydium (HEt) fluorescence between its oxidized and non-oxidized forms. The time when ɑS was applied is marked with the grey bracket on the plot. A higher increase in HEt ratio is observed upon application of ɑS solutions containing control NbHul5g, suggesting that oligomers formed in its presence are more damaging in comparison to the oligomers formed in the presence of ɑS-specific nanobodies. (TIF 108 kb) [file 12915_2017_390_MOESM8_ESM.tif]
