# Supplementary material for: Nanobodies raised against monomeric ɑ-synuclein inhibit fibril formation and destabilize toxic oligomeric species
Source: BMC Biol. 2017 Jul 3;15:57. doi: 10.1186/s12915-017-0390-6 (PMC5496350; doi:10.1186/s12915-017-0390-6)
Supplement: Supplementary file 2 — Supplementary Information. (PDF 462 kb) [file 12915_2017_390_MOESM2_ESM.pdf]

Supplementary Information

Nanobodies raised against monomeric α-synuclein inhibit fibril formation and destabilize toxic oligomeric species of α-synuclein

Marija Iljina[#], Liu Hong, Mathew H. Horrocks, Marthe H. Ludtmann, Minee L. Choi, Craig D. Hughes, Francesco S. Ruggeri, Tim Guilliams, Alexander K. Buell, Ji-Eun Lee, Sonia Gandhi, Steven F. Lee, Clare E. Bryant, Michele Vendruscolo, Tuomas P. J. Knowles, Christopher M. Dobson[*], Erwin De Genst[#*], David Klenerman[1*]

[#] Equally contributing authors.
[*]Correspondence may be addressed to:
cmd44@cam.ac.uk, erwin.degenst@astrazeneca.com, dk10012@cam.ac.uk

Supplementary Methods

Bulk ThT measurements of Alexa Fluor labeled αS. Our previous studies showed that incorporation of Alexa Fluor (AF) labels at residue 90 did not significantly affect the aggregation properties of alpha-synuclein (αS) owing to this residue being located at the periphery of the amyloid-forming region of αS[1]. Furthermore, we found that the rates of monomer incorporation into AF488 and AF594-labeled protein were the same under identical incubation conditions[2]. To confirm that AF-labeled αS assembled into fibrils over a broadly comparable timescale to unlabeled wild-type (wt) αS, we carried out bulk thioflavin-T (ThT) assays using wt and AF594-labeled protein. AF488-labeled αS was not used in this experiment because the emission form the fluorescent probe was expected to interfere with ThT fluorescence. For the assays, unlabeled 70 µM wt αS was used or 70 µM AF594-labeled A90C αS. The samples were prepared in PBS buffer (10 mM phosphate, pH 7.5, 100 mM NaCl and 0.1% NaN_3) and incubated under identical conditions, in the dark at 37 °C with agitation at 200 rpm (New Brunswick Scientific Innova 43). The aliquots were withdrawn at the same time intervals and pre-mixed with ThT solution to contain 20 µM of this dye. Measurements were performed in triplicate using Cary Eclipse spectrometer with 420 nm excitation and emission collected at 460-600 nm. Maximum ThT fluorescence intensity values from the background-corrected fluorescence spectra were plotted against incubation time (Supplementary Fig. 1a). In this experiment, the spectra of AF594-labeled αS showed an additional fluorescence emission peak centered at ~620 nm, which was well separated from the ThT emission signal and did not interfere with the measurement.

AFM Image analysis. Image flattening and statistical analysis of the average fibrillar heights was performed by SPIP (Image metrology) software, as previously described**[3-5]**. Fibrils were analyzed at a fixed resolution of 4 nm, by fitting their cross-sections to Gaussian distributions and selecting the fit maxima. Mean height of a fibril was defined as the average value of the corresponding maxima along the traced profile of the molecule. The populations of mean fibril heights are shown in Fig. 1c (main text) and Supplementary Fig. 1.

TIRFM Imaging. To confirm the inhibition of ThT-active aggregate formation during the aggregation of αS in the presence of NbSyn87 and NbSyn2, observed by bulk ThT assays (Fig. 1, main text), we performed total internal reflection fluorescence microscopy imaging (TIRFM) of unlabeled αS wt solutions according to previously reported protocols[6]. Protein

solutions were prepared and incubated under the same conditions as for the sm-FRET and bulk ThT experiments, either alone or with the addition of 2 molar equivalents of nanobodies (35 µM αS, 70 µM nanobodies, 20-h incubation). For imaging, the solutions were diluted to 200 nM (αS and αS with NbHul5g) or 500 nM (αS with NbSyn2 and αS with NbSyn87) concentration into PBS buffer (PBS, 00-3002, ThermoFisher scientific), containing 5 µM ThT and 2 nM Nile red (NR, N1142, ThermoFisher scientific), to enable the simultaneous staining of αS aggregates with ThT and NR dyes. Fluorescence imaging was carried out using custom-built TIRFM setup described previously[6]. Images were acquired at a frame rate of 20 ms and consisted of 100 frames in the ThT emission channel and 2000 frames in the NR channel. Image stacks comprising 100 frames in the ThT channel were created. The localisations in the NR channel were determined using the peak fit plugin (ImageJ, NIH, Bethesda, USA). For each sample combination, 14-19 separate image stacks were collected, enabling the characterization of 978-1815 individual protein aggregates. Representative image stacks and the quantification of the numbers of aggregates and the fractions of ThT-active species are in Supplementary Fig. 1b.

TEM Imaging. For transmission electron microscopy (TEM) imaging, 1:1 molar ratio of AF488 and AF594-labeled αS were combined either in the absence or in the presence of 2 equivalents of the studied nanobodies, and incubated under identical conditions as the samples used for sm-FRET and bulk ThT experiments. After >100-h incubation, PBS buffer was exchanged for Tris (25 mM Tris, 0.1 M NaCl, pH 7.4) using spin-filters with molecular cutoff of 5 kDa (Sartonis), because PBS buffer lead to the precipitation of negative stain in preliminary TEM imaging experiments. 10 µL volumes of the samples were applied onto carbon-coated 400-mesh copper grids (Agar Scientific) for 5 min, and washed with distilled water. Negative staining was carried out by using 2% (w/v) uranyl acetate. TEM images were acquired using Tecnai G2 microscope (13218, EDAX, AMETEK) operating at an excitation voltage of 200 kV. Representative images are shown in Supplementary Fig. 2b, and are consistent with AFM results obtained for unlabeled αS under identical incubation conditions (see main text, Fig. 1b).

QCM experiments to analyze the elongation rates of pre-formed αS fibrils in the presence of NbSyn87 and NbSyn2. Quartz crystal microbalance (QCM) measurements were carried out with E4 QCM D instrument (Q-Sense, Biolin Scientific, Stockholm, Sweden), using gold-coated QSX 301 sensor. αS fibril preparation and all subsequent measurements

were performed at 37 °C in PBS buffer, closely following previously reported protocols[7-9]. In order to outcompete the C-terminal region of αS with respect to binding to the nanobodies and regenerate the surface-bound αS fibrils, a synthetic peptide (Genemed Synthesis Inc., San Antonio, TX, USA) was used, based on the sequence of αS and comprising its residues 118-140 (118-VDPDNEAYEMPSEEGYQDYEPEA-140). QCM data are shown in Supplementary Fig. 1d. The rates of frequency decrease during the periods of incubation with αS monomer, or with an equimolar ratio of αS and NbSyn87 or NbSyn2 are included in Supplementary Tables 3 and 4, and presented as averages between the overtones N = 3,5,7. The detailed interpretation of the measured values is in Supplementary Results.

Nanobody labeling with AF dyes. Random lysine labeling was performed using N-Hydroxysuccinimde (NHS) linked AF647 or 488. 1.5 molar equivalents of AF647 (or 488) NHS ester (Life Technologies) were added to unlabeled nanobody solution in 100 mM sodium bicarbonate buffer (pH 8.3), and incubated with agitation for 3 h. The labeled protein was then separated from free dye using Sephadex G-25 loaded PD-10 desalting columns (GE Healthcare). The labeling efficiency was determined using UV-Vis absorbance measurements, and was above 90% (NanoDrop 2000c UV-Vis Spectrophotometer, Thermo Scientific). The stoichiometry of AF647 labeling of the nanobodies was determined according to the manufacturer's specifications and confirmed by mass-spectrometry and was found to be close to a 1:1 stoichiometry. The presence of the dye did not interfere with αS binding as observed using total internal reflection fluorescence spectrometry and SPR (data not shown).

TCCD data acquisition and analysis. To verify the absence of binding between fluorescently-labeled nanobodies and fluorescently-labeled αS at single-molecule concentrations in our experiments, control two-color coincidence detection (TCCD) was performed. Dual excitation in either 488/594 nm or 488/633 nm mode (depending on the AF label pairs of the analyzed samples) was used, and single-molecule confocal instrumentation and methodology as previously described in detail[10, 11], utilizing a detection under fast-flow as for the sm-FRET measurements. In TCCD experiments, in contrast to monitoring FRET signal between the dye pairs, direct fluorescence signal from both AF488 and AF594 (or AF647) was detected as a consequence of direct dual-color laser excitation. Bound species bearing two different fluorophores are expected to produce two fluorescence bursts that are coincident in time, while singly labeled molecules will produce non-coincident

fluorescence bursts. The coincidence can be quantified using the association quotient Q, defined as:

$$Q = \frac{C - E}{A + B - (C - E)} \quad \text{(S1)}$$

where A is the number of fluorescent bursts in the blue channel above the 15 photons bin^{-1} threshold, B is the number of fluorescent bursts in the red channel, C is the number of coincident events, and E is the number of chance-coincident events. Q value arising purely due to chance coincidence events was determined by analyzing free AF dyes in solution, as described below.

For the measurements, 1:1 stoichiometric ratio of AF-labeled αS and NbSyn87 was used. This nanobody was chosen owing to its highest affinity for αS and therefore the highest potential chance for it to remain bound in our single-molecule experiments. The following equimolar combinations were tested: AF647-NbSyn87 + AF488-αS, AF488-NbSyn87 + AF647-αS, AF488-NbSyn87 + AF594-αS. In addition, samples of free AF647 + AF488 and aggregated AF647-αS + AF488-αS were analyzed as controls. For the measurements, the samples were diluted either in PBS buffer or deionized water up to single-molecule concentration suitable for the TCCD analysis. The dilution into different buffers served as a test for potential changes in the co-interaction of the nanobody and αS due to the changes in pH and ionic strength. Following the dilution, solutions were immediately introduced into a straight channel of a microfluidic device via a gel-loading tip (200 µL, Life technologies) and withdrawn at a constant rate of 1 cm s^{-1} via a syringe pump (PHD2000, Harvard Apparatus). Overlapped laser beams were focused into the middle of the channel. For each sample, data were acquired for 600 s, with 100 µs bin-width, chosen according to the expected residence time in the excitation region at the chosen flow speed[12], 100,000 bins per frame and a total of 60 frames. The fluorescence photon traces in two separate channels, the emission from AF488 and AF594 dyes (or AF647, when the samples and the setup were in 488/647 mode), were collected simultaneously and outputted using custom-programmed field-programmable gate array (FPGA, Colexica). All measurements were made at ambient temperature around 20 °C, similarly to sm-FRET measurements.

The collected photon traces were analyzed using custom-written Igor Pro 6.22 (Wavemetrics) software analogous to previously described[2]. The data were corrected for autofluorescence and the crosstalk. Photon bursts with intensities greater than the threshold of 15 $photons.bin^{-1}$ in the blue and in the red channels were selected according to previously established

threshold selection approach that allows maximizing the detection of coincident events. Simultaneous events in both channels above the threshold (the AND criterion[13]) were selected. To account for any possible coincident events due to chance, the desynchronization approach was used[14]. Time-bins in the blue channel were randomly re-numbered before the selection of simultaneous events in the two channels above the threshold. Using these outputs, the association quotient Q was estimated according to equation S1. The resulting values are summarized in Supplementary Table 1.

TCCD chance coincidence controls using free dyes in solution or dual-labeled αS. To determine Q corresponding to a non-interacting system and resulting purely from the chance-coincidence, 1:1 stoichiometric mixture of free AF dyes were measured by TCCD, keeping the same detection conditions as for the protein measurements. The free unbound dyes were prepared according to a previously published protocol[15]. For the measurement, 1:1 molar ratio of AF488 and AF647 were used. The samples were recorded straight after preparation. As positive controls, the samples of dual-labeled αS incubated under agitation at 2 µM for 72 h were also recorded. The data were analyzed in the same manner as the data recorded using the fluorescent proteins and the resulting Q values are presented in Supplementary Table 1.

In all cases, the Q values from NbSyn87 + αS samples were comparable to the values obtained for the free dyes in solution, and lower than the Q value for aggregated αS. Therefore, it can be concluded that the nanobody is fully unbound from αS in the single-molecule experiments. This result suggests that the observed differences in FRET histograms in single-molecule experiments are not due to photophysical effects on the fluorophores caused by bound nanobodies and supports the hypothesis that they are due to the structural differences of the analyzed oligomers.

Comparison of average αS monomer fluorescence intensities in the presence or in the absence of unlabeled nanobodies. As an additional test to verify that the unlabeled nanobodies did not interfere with the fluorescence emission from AF labels on αS, the values of average monomer brightness in sm-FRET measurements were compared across the samples with or without the nanobodies, following data collection with the sample dilutions in either PBS or water. The resulting average AF488-αS monomer intensities selected above the threshold of 15 photons.bin^{-1} are listed in Supplementary Table 2. The values are all

comparable, additionally confirming the absence of alteration of fluorescence signal in the presence of nanobodies.

Kinetic analysis. The kinetic analysis was as recently reported[16], with the additional inclusion of two explicit reverse reactions from high-FRET oligomers to low-FRET oligomers, and from low-FRET oligomers to monomers, as shown in Supplementary Fig. 3a.

The following kinetic moment equations were used to model the aggregation of αS in the absence and in the presence of nanobodies:

$$\begin{cases} \dot{O}_l(t) = k_n m(t)^{n_c} - \widetilde{k_n} O_l(t) - k_1^c O_l(t) + \widetilde{k_1^c} O_h(t), \\ \dot{O}_h(t) = k_1^c O_l(t) - \widetilde{k_1^c} O_h(t) - k_2^c O_h(t), \\ \dot{P}(t) = k_2^c O_h(t), \\ \dot{M}(t) = n_h k_2^c O_h(t) + 2k_e m(t) P(t), \\ m(t) = m_{tot} - n_l O_l(t) - n_h O_h(t) - M(t). \end{cases} \quad \text{(S2)}$$

where $O_l(t)$ and $O_h(t)$ are the number concentration of low-FRET and high-FRET oligomers, $P(t)$ and $M(t)$ are the number and mass concentration of fibrils, $m(t)$ is the concentration of free αS monomer units, m_{tot} is the total concentration of monomer in the system, n_l and n_h are the average size of low-FRET and high-FRET oligomers, which for simplicity are both set equal to n_c the critical nucleus size for primary nucleation. $k_n, k_1^c, k_{2,}^c\ k_e$ are the rate constants governing the primary nucleation of low-FRET oligomers, the conversion from low-FRET oligomers to high-FRET oligomers, the conversion from high-FRET oligomers to fibrils, and the growth of fibrils respectively. Two reverse reactions, with the rate constants $\tilde{k}_n$ and $\tilde{k}_1^c$ are introduced in order to describe the inhibitory effects of nanobodies. In Fig. 3b-e, global fits are performed with the above model using four experimental data sets with and without nanobodies. Parameters are determined through the best fit to fibril, monomer and oligomer kinetics simultaneously. Error estimations are carried out on each parameter separately by keeping the relative difference of all predictions within 20%.

Surface Plasmon Resonance Measurements. The kinetic constants of the interaction of NbSyn87 with wt αS were determined by Surface Plasmon Resonance (SPR) measurements

(Biacore 3,000, GE Healthcare, Sweden). The αS protein was immobilized on a CM5 chip at approximately 50 resonance units (50 pg/mm2), via EDC/NHS chemistry according to the manufacturer's recommendations. Triplicate association traces of NbSyn87 for concentrations ranging from 0 µM to 227 µM were recorded for 2 min in a buffer containing 10 mM HEPES pH 7.4, 150 mM NaCl, 0.005 % Tween 20 and 3 mM EDTA, at 25 °C. Triplicate dissociation traces of the complexes in the buffer were recorded for 5 min. Curves obtained after subtraction of the signals of the reference cell (the flow cell that contained no immobilized protein) and injection of buffer, were then fitted to a 1:1 Langmuir binding model with the program BIAeval (Biacore). The resulting values for NbSyn87: αS interaction were: $k_{on} = (4.09 \pm 0.04) \times 10^6\ M^{-1}s^{-1}$; $k_{off} = (32.0 \pm 0.1) \times 10^{-3}\ s^{-1}$; and $K_d=(7.82 \pm 0.08) \times 10^{-9}$ M. These values were expected to increase but stay within the same order of magnitude at 37 °C (El Turk *et. al.*, Manuscript in preparation). For the NbSyn2: αS interaction, the kinetics were considered too fast to be measured reliably by SPR. If to assume that the k_{on} is in the order of $1\times10^6\ M^{-1}s^{-1}$, then $k_{off} = Kd*k_{on} = 0.26\ s^{-1}$ for a Kd value of 260 nM at 37 °C[17].

Proteinase K digestion assays. To confirm the structural differences of αS oligomers generated in the presence of αS-specific nanobodies to the oligomers formed in the presence of the control nanobody, their susceptibility to proteinase K digestion was measured. The sample preparation was the same as for sm-FRET kinetic measurements, and solutions contained a 1:1 dual-labeled mixture of αS (70 µM) with 2 equivalents of unlabeled nanobodies. Proteinase K aliquots were prepared in PBS buffer of the same composition as in all previous experiments, with the addition of 1 mM $CaCl_2$, and stored at -80 °C before use. Protein samples after 29-h incubation under the same conditions as in previous experiments were diluted into a range of proteinase K concentrations between 0-10 µg ml^{-1} in PBS buffer with 1 mM $CaCl_2$. The total protein concentration during incubations with proteinase K was 280 nM of αS and 560 nM of nanobodies. Note that even though only fluorescently labeled αS was detected in this experiment and unlabeled nanobodies remained 'invisible', both αS and nanobodies were expected to be susceptible to the digestion by proteinase K. Therefore, αS samples in the absence of nanobodies were not measured to ensure consistent comparison between the same starting total protein concentrations. The samples were incubated at 37 °C for 10 min, and subsequently further diluted (up to 280 pM of αS) prior to analysis by sm-FRET. The results, presented as a fraction of degradation against proteinase K

concentrations, are in Fig. 4a (main text), and representative data obtained for individual timepoints are in Supplementary Fig. 5. To note, the relative minor difference in profiles of the samples aggregated in the presence of αS-specific nanobodies (Fig. 4a, main text) may reflect the differences in the binding affinity of the two nanobodies rather than the structural dissimilarity of the αS oligomers, since the samples containing both nanobodies yielded similar FRET efficiency distributions (Supplementary Fig. 5). The binding of αS-specific nanobodies may protect against αS degradation. Because of the 10-fold difference in the affinities of the two αS-specific nanobodies, higher fraction of αS was expected to be bound by NbSyn87 than by NbSyn2 under the proteinase K incubation conditions, hence explaining the resulting higher stability of oligomers formed in the presence of NbSyn87 in comparison to the oligomers formed in the presence of NbSyn2 in this assay.

Proteasome degradation assays. These assays, presented in Fig. 4b (main text) were carried out according to previously described protocol[18]. For the comparison between the nanobody-containing samples and αS-only samples after 29 hours of incubation with respect to their stability towards degradation by 26S proteasome, the samples were diluted for proteasomal degradation to contain 40 nM proteasome, 125 mM ATP.$MgCl_2$, 5 µM creatine kinase and 0.1 M creatine phosphatase in 50 mM Tris buffer (pH 7.4). The resulting samples were analyzed by sm-FRET both immediately after mixing, and after incubation for 24 h under quiescent conditions at 37 °C. The fractions of non-degraded oligomers were determined as the numbers of oligomers after the incubation divided by the numbers of oligomers immediately after mixing (Fig. 4b, main text).

ROS measurements. Mixed cultures of hippocampal or cortical rat neurons and glial cells were prepared and cultured as described previously[19]. The experiments were carried out according to previously detailed protocols[1, 20]. For the assays presented in Fig. 4c (main text), AF647-αS was used because the spectral properties of AF647 were more compatible with the experiment than of AF488 or AF594 dyes. 70 µM samples of αS, either in the absence or presence of 2 equivalents of NbSyn2, NbSyn87 and NbHuL5g, were prepared and incubated under identical conditions as for sm-FRET experiments. After 29-h incubation, the solutions were withdrawn and used for ROS experiments. The solutions were applied to

primary co-cultures of neurons and astrocytes loaded with dihydroethidium (HEt) (2 μM HEt was present in the solution during the experiments). No preincubation ("loading") with HEt was used in order to limit the intracellular accumulation of oxidized products. For HEt measurements, a ratio of the fluorescence intensities resulting from its oxidized/reduced forms was quantified. Emission recorded above 560 nm was assigned to be from the oxidized form (ethidium), while emission collected from 405 to 470 nm was from the reduced form (hydroethidium). The rate of ROS production was determined by dividing the gradient of the HEt ratio function against time after application of 500 nM αS solution by the gradient prior to αS application. The representative plots of HEt ratio against time are shown in Supplementary Fig. 6. Consistent with our previous reports[1, 20], application of 500-nM αS induced an increase in the rate of ROS production. Application of αS that had been aggregated in the presence of control NbHul5g resulted in a similar increase in ROS production, with no significant difference between the response caused by αS and αS plus NbHul5g. In contrast, the application of αS sample aggregated with NbSyn87 as well as the application of αS aggregated with NbSyn2 lead to significantly reduced rate of ROS (Fig. 4c, main text).

Toxicity (cell death) experiments. These assays, presented in Fig. 4d (main text), were carried out to investigate whether the effect of the nanobodies on the aggregation process reduced the ability of αS aggregates to induce cell death. We applied the αS solutions prepared as before, either in aqueous buffer or in the presence of nanobodies, to primary co-cultures for 4 h. The cells were loaded simultaneously with 20 μM propidium iodide (PI), which is excluded from viable cells but exhibits red fluorescence following a loss of membrane integrity, and 4.5 μM Hoechst 33342 (Molecular Probes), which gives blue staining to chromatin, to count the total number of cells. A total number of 600-800 neurons or glial cells were counted in 10 fields of each coverslip after the 4-h incubation with 100-nM αS samples that had been aggregated in the presence or the absence of the various nanobodies. Each experiment was repeated 5 or more times using separate cultures. The incubation of cells with 100-nM αS samples for 4 h resulted in an increase in cell death, while the incubation of cells with αS aggregated in the presence of either NbSyn87 or NbSyn2 lead to a significant reduction in cell death (Fig. 4d). Incubation of cells with either nanobodies in the absence of αS was confirmed not to alter the basal rate of cell death.

Quantification of TNF-α production. The experiments in Fig. 4e (main text) were carried out using BV2 microglia, according to the previously reported protocol[18]. αS-only (wt) at 70 µM, and in the presence of 140 µM of nanobodies were prepared as for all previous aggregation assays, withdrawn at 29 h and applied at 10 µM concentration of αS. After application, the cells were incubated at 37 °C for 24 h, and the supernatants were subsequently collected and analyzed using TNF-α Elisa kit (R & D, Minneapolis, MN) according to manufacturer's protocol.

Statistical analysis of the data from cell experiments. Student t-tests (two-tailed) were carried out using Origin 9 software (Microcal Software) or Microsoft Excel, and the resulting ranges of p values are included in Fig. 4 (main text).

Supplementary Results

Analysis of QCM experiments: elongation of αS fibrils in the presence of NbSyn87 and NbSyn2.

The data for both nanobodies show that the elongation rate is reduced directly after incubation with nanobodies, or, even more clearly, in the presence of nanobodies. In both cases the reduction in measured rate of frequency change is ca. 50%. Given that stoichiometric amounts of αS monomer and nanobody are added, the elongation likely proceeds through the addition of αS-nanobody complex. This complex has a mass of the order of 24 kDa, compared to the molecular mass of αS alone of ca. 14 kDa. This difference is relevant, as QCM senses the change in mass of the surface-bound material. If the signal in the QCM experiment would only stem from the dry mass of the added protein, a decrease of the rate of frequency change by 50% would translate therefore into an inhibition of 70%, representing an upper bound of the observed inhibition.

We have shown in the past that the mass sensitivity of QCM for fibril growth is ca. 4 times as high as predicted by the Sauerbrey equation[8], using the dry mass. Therefore, only ca. 25% of the signal stem from the dry mass, and 75% stem from water that is dragged along with the fibrils. If the frequency shift were proportional to the dry mass, i.e. that the dry mass always contributes the same fraction to the frequency shift, then this would yield the same degree of inhibition as calculated above (70%). If, however, we assume that the water contribution is independent of the mass of the elongating unit (and rather defined by the fibril geometry, which is probably not strongly dependent on the binding of the nanobody), we obtain a lower bound for inhibition, corresponding to 58%.
Therefore, based on the QCM experiments, the elongation rate of pre-formed αS fibrils in the presence of nanobody corresponds to 30-40% of the rate in the absence of nanobodies.

Kinetic analysis of αS aggregation in the presence of nanobodies.

Our experimental results showed that the presence of both αS-specific nanobodies slowed down the aggregation of αS. Using sm-FRET measurements, we characterized the impact of nanobodies on the earliest steps of the aggregation reaction and observed a clear inhibition of high-FRET oligomer formation. To quantitatively characterize this inhibition in the presence of the nanobodies, we applied kinetic analysis, using the nucleation-conversion-

polymerization model that we have recently reported for the aggregation of αS alone[16], but additionally including two explicit reverse reactions, as is schematically outlined in Supplementary Fig. 3a. According to this model, monomeric αS molecules assemble into low-FRET oligomers, which subsequently convert into high-FRET oligomers. These in turn convert into fibrils, which then grow via a succession of monomer addition steps. As previously[16], within this description, oligomer conversion and fibril elongation steps are treated as size-independent, and any late-stage processes such as fibril fragmentation do not enter the analysis. To account for the observed inhibitory effects of the nanobodies on oligomer conversion, we introduced two explicit reverse reaction steps, from high-FRET oligomers to low-FRET oligomers and from low-FRET oligomers to monomers. The inclusion of these steps enabled the overall rate of conversion to high-FRET oligomers to be reduced in the presence of the αS-specific nanobodies, assuming the forward reactions to be unaltered. It was confirmed that the addition of these two steps was necessary for the analysis, because either decreasing the rates of forward reactions or solely decreasing one of the two reverse reaction rates resulted in large deviations between the predicted and observed ranges of aggregate concentrations.

We used the modified model to globally fit the kinetic profiles of monomer depletion, oligomer formation and fibril formation. The rate constants reported in Supplementary Fig. 3 result from the best fit to monomer, oligomer and fibril kinetics simultaneously, and the fits are shown in Supplementary Fig. 3b-e. All resulting parameters of the forward reactions are consistent with our previous results[23]. The estimated rate constants for the conversion from high-FRET to low-FRET oligomers, $\tilde{k}_1^c$, were 1± 0.5 h^{-1} for NbSyn2 and 10±5 h^{-1} for NbSyn87, and both values are consistent with our independent experimental observation of the rapid conversion within minutes of pre-formed high-FRET oligomers to low-FRET oligomers upon the addition of nanobodies (shown in main text, Fig. 3). Using the derived parameters, we predicted the kinetics of low-FRET and high-FRET oligomer production during the aggregation reaction over longer time-period up to 100 h. According to these predictions shown in Supplementary Fig. 3f, the concentration of low-FRET oligomers would decrease slower in the presence of the αS-specific nanobodies than in their absence. Furthermore, the formation of high-FRET oligomers would be inhibited, in good agreement with the sm-FRET findings. These effects are predicted to be stronger in the presence of NbSyn87, consistent with its higher affinity for αS in comparison to NbSyn2.

Supplementary Tables

Supplementary Table 1. Summary of %Q values (eq. S1), resulting from TCCD control experiments using 1:1 stoichiometric ratio of fluorescently labeled NbSyn87 and αS. Free dyes are measured as a negative control, and aggregated dual-labeled αS as a positive control. Buffers used for the dilutions are specified.

	Free AF 488 + AF647	αS, 2 µM, 72 h	AF647-NbSyn87 + AF488-αS		AF488-NbSyn87 + AF647- αS		AF488-NbSyn87 + AF594-αS
	**PBS**	**PBS**	**PBS**	**H_2O**	**PBS**	**H_2O**	**PBS**
**%Q**	0.025 ± 0.030	0.917 ± 0.291	0.093 ± 0.13	0.094 ± 0.001	0.012 ± 0.029	0.051 ± 0.051	0.021 ± 0.010

Supplementary Table 2. Average AF488-αS monomer intensity above the threshold of 15 photons.bin^{-1}, as determined from sm-FRET measurements ($N_{molecules}$ = 185,584).

Sample type	αS		αS+ NbHul5g		αS+ NbSyn2		αS+ NbSyn87	
	**PBS**	**H_2O**	**PBS**	**H_2O**	**PBS**	**H_2O**	**PBS**	**H_2O**
**Mean intensity** (photons. bin^{-1})	19.4	19.2	19.2	19.0	19.3	19.1	19.2	19.4

Supplementary Table 3. QCM experimental results. The rates of frequency decrease during the periods of incubation with αS monomer, or in the presence of NbSyn87, are reported as averages between the overtones N = 3,5,7.

**Condition**	21 µM αS	21 µM αS	21 µM αS	21 µM αS	21 µM αS + 21 µM NbSyn87	21 µM αS
**Time**	0:07	0:24	1:25	1:43	2:24	3:09
**Rate (Hz min^{-1})**	-3.9447	-4.3306	-2.3611	-3.5209	-1.6863	-2.6857

Supplementary Table 4. QCM experimental results. The rates of frequency decrease during the periods of incubation with αS monomer, or in the presence of NbSyn2 or NbHul5g, again, reported as averages between the overtones N = 3,5,7.

**Condition**	21 µM αS	21 µM αS	21 µM αS + 21 µM NbSyn2	21 µM αS	21 µM αS + 21 µM NbHul5g	21 µM αS + 21 µM NbSyn2	21 µM αS
**Time**	0:33	0:43	1:00	1:25	2:03	2:24	3:09
**Rate (Hz min^{-1})**	-3.7594	-3.8293	-1.9180	-4.4687	-3.8466	-2.1205	-3.7981

Supplementary References

1. Cremades N, Cohen SI, Deas E, Abramov AY, Chen AY, Orte A, Sandal M, Clarke RW, Dunne P, Aprile FA, et al: Direct observation of the interconversion of normal and toxic forms of α-synuclein. Cell. 2012;149:1048-1059.
2. Horrocks MH, Tosatto L, Dear AJ, Garcia GA, Iljina M, Cremades N, Dalla Serra M, Knowles TP, Dobson CM, Klenerman D: Fast Flow Microfluidics and Single-Molecule Fluorescence for the Rapid Characterization of α-Synuclein Oligomers. Anal Chem. 2015;87:8818-8826.
3. Ansaloni A, Wang ZM, Jeong JS, Ruggeri FS, Dietler G, Lashuel HA: One-pot semisynthesis of exon 1 of the Huntingtin protein: new tools for elucidating the role of posttranslational modifications in the pathogenesis of Huntington's disease. Angew Chem Int Ed Engl. 2014;53:1928-1933.
4. Khalaf O, Fauvet B, Oueslati A, Dikiy I, Mahul-Mellier AL, Ruggeri FS, Mbefo MK, Vercruysse F, Dietler G, Lee SJ, et al: The H50Q mutation enhances α-synuclein aggregation, secretion, and toxicity. J Biol Chem. 2014;289:21856-21876.
5. Ruggeri FS, Habchi J, Cerreta A, Dietler G: AFM-Based Single Molecule Techniques: unraveling the Amyloid Pathogenic Species. Curr Pharm Des. 2016.
6. Bongiovanni MN, Godet J, Horrocks MH, Tosatto L, Carr AR, Wirthensohn DC, Ranasinghe RT, Lee JE, Ponjavic A, Fritz JV, et al: Multi-dimensional super-resolution imaging enables surface hydrophobicity mapping. Nat Commun. 2016;7:13544.
7. Buell AK, White DA, Meier C, Welland ME, Knowles TP, Dobson CM: Surface attachment of protein fibrils via covalent modification strategies. J Phys Chem B. 2010;114:10925-10938.
8. Michaels TC, Buell AK, Terentjev EM, Knowles TP: Quantitative Analysis of Diffusive Reactions at the Solid-Liquid Interface in Finite Systems. J Phys Chem Lett. 2014;5:695-699.
9. Brown JW, Buell AK, Michaels TC, Meisl G, Carozza J, Flagmeier P, Vendruscolo M, Knowles TP, Dobson CM, Galvagnion C: β-Synuclein suppresses both the initiation and amplification steps of α-synuclein aggregation via competitive binding to surfaces. Sci Rep. 2016;6:36010.
10. Orte A, Birkett NR, Clarke RW, Devlin GL, Dobson CM, Klenerman D: Direct characterization of amyloidogenic oligomers by single-molecule fluorescence. Proc Natl Acad Sci U S A. 2008;105:14424-14429.
11. Narayan P, Orte A, Clarke RW, Bolognesi B, Hook S, Ganzinger KA, Meehan S, Wilson MR, Dobson CM, Klenerman D: The extracellular chaperone clusterin sequesters oligomeric forms of the amyloid-β(1-40) peptide. Nat Struct Mol Biol. 2012;19:79-83.
12. Horrocks MH, Li H, Shim JU, Ranasinghe RT, Clarke RW, Huck WT, Abell C, Klenerman D: Single molecule fluorescence under conditions of fast flow. Anal Chem. 2012;84:179-185.
13. Ying L, Wallace M, Balasubramanian S, Klenerman D: Ratiometric analysis of single-molecule fluorescence resonance energy transfer using logical combinations of threshold criteria: A study of 12-mer DNA. Journal of Physical Chemistry B. 2000;104:5171-5178.

14. Clarke R, Orte A, Klenerman D: Optimized threshold selection for single-molecule two-color fluorescence coincidence spectroscopy. Analytical Chemistry. 2007;79:2771-2777.
15. Rey NL, Petit GH, Bousset L, Melki R, Brundin P: Transfer of human α-synuclein from the olfactory bulb to interconnected brain regions in mice. Acta Neuropathol. 2013;126:555-573.
16. Iljina M, Garcia GA, Horrocks MH, Tosatto L, Choi ML, Ganzinger KA, Abramov AY, Gandhi S, Wood NW, Cremades N, et al: Kinetic model of the aggregation of alpha-synuclein provides insights into prion-like spreading. Proc Natl Acad Sci U S A. 2016.
17. De Genst EJ, Guilliams T, Wellens J, O'Day EM, Waudby CA, Meehan S, Dumoulin M, Hsu ST, Cremades N, Verschueren KH, et al: Structure and properties of a complex of α-synuclein and a single-domain camelid antibody. J Mol Biol. 2010;402:326-343.
18. Iljina M, Tosatto L, Choi ML, Sang JC, Ye Y, Hughes CD, Bryant CE, Gandhi S, Klenerman D: Arachidonic acid mediates the formation of abundant alpha-helical multimers of alpha-synuclein. Sci Rep. 2016;6:33928.
19. Vaarmann A, Gandhi S, Gourine AV, Abramov AY: Novel pathway for an old neurotransmitter: dopamine-induced neuronal calcium signalling via receptor-independent mechanisms. Cell Calcium. 2010;48:176-182.
20. Deas E, Cremades N, Angelova PR, Ludtmann MH, Yao Z, Chen S, Horrocks MH, Banushi B, Little D, Devine MJ, et al: Alpha-Synuclein Oligomers Interact with Metal Ions to Induce Oxidative Stress and Neuronal Death in Parkinson's Disease. Antioxid Redox Signal 2016;24:376-391.
